# Homoallylic amines by reductive inter- and intramolecular coupling of allenes and nitriles

**DOI:** 10.3762/bjoc.7.94

**Published:** 2011-06-17

**Authors:** Peter Wipf, Marija D Manojlovic

**Affiliations:** 1 Department of Chemistry, University of Pittsburgh, 219 Parkman Avenue, Pittsburgh, PA 15260, USA

**Keywords:** allene, 3-aminotetrahydrofurans, 3- and 4-aminotetrahydropyrans, hydrozirconation, nitrile, transmetalation

## Abstract

The one-pot hydrozirconation of allenes and nitriles followed by an in situ transmetalation of the allylzirconocene with dimethylzinc or zinc chloride provides functionalized homoallylic amines. An intramolecular version of this process leads to 3-aminotetrahydrofurans and 3-aminotetrahydropyrans.

## Introduction

The reversible addition of zirconocene hydrochloride (Cp_2_Zr(H)Cl, Schwartz’s reagent) to π-bonds usually leads predominantly to σ-complexes, and the resulting organozirconocene complexes are valuable reactive intermediates for the formation of carbon–halogen and carbon–carbon bonds [1–6]. The reaction of Schwartz’s reagent with allenes occurs at low temperature and provides a ready access to σ-bound allylzirconocenes [7]. These species can be added diastereoselectively to aldehydes and ketones to yield homoallylic alcohols, but they are generally not sufficiently reactive towards many other electrophiles [8–9]. Similar to the related alkyl- and alkenyl- zirconocenes [1–3,6], this limitation of sterically hindered allylzirconocene complexes can be overcome by selective transmetalation of zirconium to other metals. Suzuki and co-workers treated allylzirconocenes with methylaluminoxane (MAO) in order to achieve the carbalumination of 1-alkynes [10], internal alkynes [11], conjugated enynes [12], and 1-iodoalkynes [13]. Huang and Pi found that allylzirconocenes underwent conjugated addition to enones in the presence of CuBr·SMe_2_ [14]. Wipf and Pierce demonstrated that, upon the addition of a zinc reagent to allylzirconocenes, transient allylzinc intermediates can be successfully added to phosphoryl- and sulfonylimines to provide homoallylic amines in good yields and diastereoselectivities [15]. Of particular interest was the reaction of tin- or silicon-substituted allenes that furnish bis-metallic reagents that could potentially serve as dianion equivalents and provide (*E*)-vinylsilanes and (*E*)-vinylstannanes in good yields [15].

*N*-Metalloimines are reactive intermediates that represent masked imine derivatives of ammonia, which are often unstable and difficult to prepare. A common method for the preparation of these species is the addition of various metal hydrides to nitriles [16–20], including aluminium [21–24], niobium [25], samarium [26] and iron hydrides [27]. Zirconocene hydrochloride can also be added to nitriles to provide *N*-zirconoimines, which can be trapped with a range of electrophiles to form imine derivatives [28–30]. Floreancig and co-workers developed a method for the preparation of α-functionalized amides by trapping *N*-zirconoimines with acyl chlorides, followed by the addition of nucleophiles to the intermediate acyl imines [31–33]. Furthermore, the utility of the hydrozirconation of nitriles can be enhanced by using Lewis acids to engage nitrile-derived acylimines in Friedel–Crafts reactions, generating indanyl or tetrahydronaphthyl derivatives [34–35].

Previous work in our group had concentrated on the transmetalation of alkenyl- and allylzirconium species to give zinc organometallics, which were added to phosphoryl- and sulfonylimines to obtain homoallylic amines [15,36]. The preparation of phosphoryl- and sulfonylimines as well as the subsequent removal of these activating groups was often low-yielding. Because of that, as well as limited functional group compatibility in this methodology, we sought to develop a new approach for the protective group-free synthesis of homoallylic amines. The ease of synthesis of *N*-metalloimines by hydrometalation of nitriles could potentially provide suitable intermediates for this synthetic strategy. In this article, we report a one-pot hydrozirconation of allenes and nitriles that facilitates the reductive coupling to yield *N*-unprotected homoallylic amines.

## Results and Discussion

We first investigated the addition of allylzirconocenes to *N-*aluminoimines. *N*-aluminoisobutyroimine **1** was prepared in situ by the reduction of nitrile **3** with DIBAL (1 equiv) in toluene. The resulting mixture was cannulated at −78 °C into a solution of allylzirconocene (1.4 equiv), prepared by the hydrozirconation of 3-methyl-1,2-butadiene (**2**). After stirring for 30 min, the desired product **4** was isolated in 76% yield as a single regioisomer. Other aliphatic nitriles were also good substrates for this reaction; however, *N*-aluminoimines obtained from aromatic nitriles were unreactive towards allylzirconocenes, and the desired product was not detected from these substrates (Table 1).

**Table 1:** Reaction of *N*-aluminoimines with allylzirconocene derived from allene **2**.

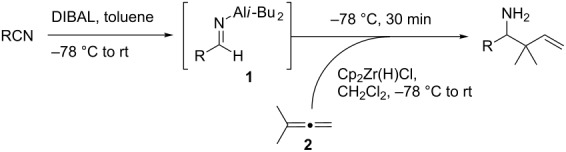			

Entry	Nitrile	Product	Yield (%)

1	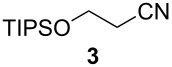	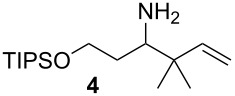	76
2	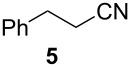	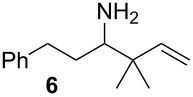	69
3	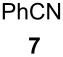	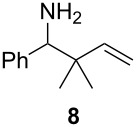	—^a^

^a^None of the desired product was detected in this reaction; instead, a mixture of high molecular weight byproducts was observed.

**Scheme 1:**
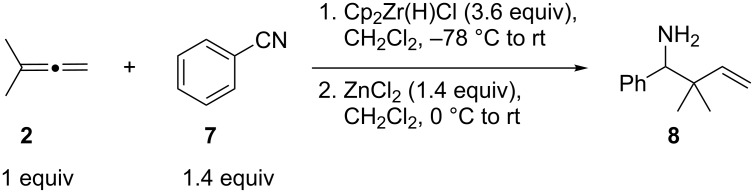
One-pot hydrozirconation-reductive coupling of allene **2** and nitrile **7**.

We also investigated the one-pot hydrozirconation of allenes and nitriles, aiming to explore the in situ formation-addition of allylzirconocenes to *N*-zirconoimines (Scheme 1). Exposure of benzonitrile (**7**) and 3-methyl-1,2-butadiene (**2**) to an excess of Schwartz’s reagent in CH_2_Cl_2_ at −78 °C led to the formation of a bright red solution after gradual warming to room temperature. However, upon aqueous work-up, none of the desired amine was obtained, even when the more Lewis acidic Cp_2_Zr(H)Cl prepared in situ by the Negishi protocol [37] was used. In contrast, adding 1.4 equiv of ZnCl_2_ to the hydrozirconation reaction mixture, according to Suzuki’s protocol for the reductive coupling of allenes and alkynes [38], led to the formation of homoallylic amine **8** in 75% yield after stirring at room temperature for 3 h.

Under the optimized conditions for the reaction of benzonitrile (**7**), we further explored the scope of the reaction of nitriles with **2** (Table 2). Both aromatic (Table 2, entries 1 and 2) and aliphatic (Table 2, entries 3 and 4) *N*-zirconoimines derived from the corresponding nitriles reacted smoothly with allylzirconocene in the presence of a slight excess of ZnCl_2_ (1.4 equiv) to give homoallylic amines in moderate to good yields. The phenylallene **13** yielded exclusively the terminal alkene product **15** in good yield as a single diastereoisomer. In all of these examples, the γ-adduct was isolated as the sole regioisomer, and no internal alkene was detected. This regioselectivity is consistent with the allylzincation of imines [15] and opposite to that of the zinca-Claisen reaction observed by Suzuki and co-workers [38]. Analogous to the previous work in our group [15], the silyl-substituted allene **16** produced the (*E*)-vinylsilane **17** as the sole product in this reaction.

**Table 2:** Reductive coupling of allenes and nitriles in the presence of Cp_2_Zr(H)Cl and ZnCl_2_.^a^

Entry	Allene	Nitrile	Product	Yield (%)

1	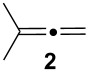	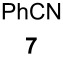	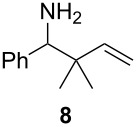	75 (78)^b^
2	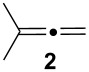	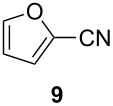	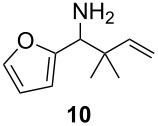	55 (67)^b^
3	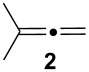	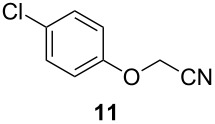	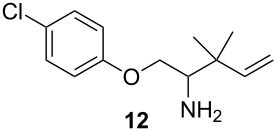	80 (81)^b^
4	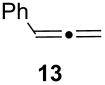	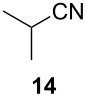	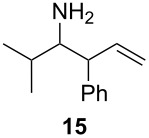	65 (71)^b,c^
5	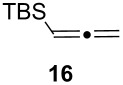	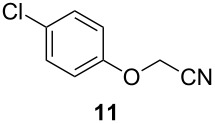	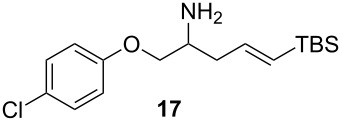	67 (70)^b,d^

^a^All reactions were carried out by hydrozirconation of a mixture of allene (1.4 equiv) and nitrile (1 equiv) in CH_2_Cl_2_ at −78 °C, followed by the addition of a 1 M solution of ZnCl_2_ in ether (1.4 equiv) at 0 °C. ^b^Yields in parentheses correspond to the reaction in which transmetalation was performed using Me_2_Zn (1.4 equiv) in toluene. ^c^Only one diastereoisomer was observed by ^1^H NMR analysis of the crude reaction mixture. ^d^Alkene geometry was assigned by coupling constant analysis.

**Scheme 2:**
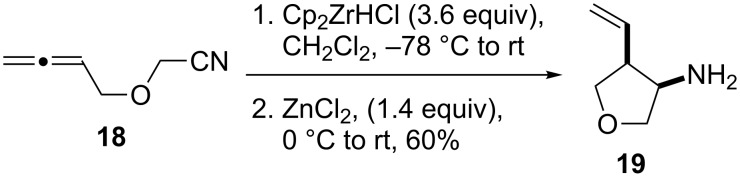
Cyclization of allenylnitrile **18**.

Given the success of the one-pot intermolecular reductive coupling of allenes and nitriles, we sought to expand our methodology to an intramolecular variant. For this purpose, we synthesized substrate **18** by *O*-alkylation of allenylmethanol [39] with bromoacetonitrile (Supporting Information File 1). We were pleased to see that treatment of substrate **18** with 3.6 equiv of Schwartz’s reagent in CH_2_Cl_2_ followed by the addition of 1.4 equiv of ZnCl_2_ led to the formation of the desired tetrahydrofuran product **19** as a single diastereoisomer in 60% yield (Scheme 2). Surprisingly, however, we found that repeating this reaction gave variable yields. Because the reaction mixture was heterogeneous after the addition of the zinc salt, we argued that decreasing the amount of Cp_2_Zr(H)Cl or a lower substrate concentration might help to address this problem. Unfortunately, these studies were inconclusive, and 3.6 equiv of Schwartz’s reagent were generally needed for a satisfactory reaction progress.

To address the reproducibility issue, we also investigated different zinc sources (Table 3). The presence of zinc halides and triflates (ZnCl_2_, Zn(OTf)_2_) in a range of solvents always resulted in the formation of a precipitate. Therefore, we turned our attention to dialkylzincs. We were pleased to see that the addition of a 1 M solution of diethylzinc to the hydrozirconated **18** at −78 °C produced a homogeneous red solution; however, only traces of the desired product **19** were detected. Switching the solvent from CH_2_Cl_2_ to toluene before the addition of the dialkylzinc reagent resulted in the desired product formation in good yield and provided a single diastereoisomer. This observation is in agreement with previous work in our group that showed the transmetalation from zirconium to zinc to occur faster in toluene than in CH_2_Cl_2_ [15]. Furthermore, we also repeated some earlier examples of the intermolecular reaction using dimethylzinc in toluene for the transmetalation step. These new experiments produced results similar to the reactions in the presence of zinc chloride (Table 2).

**Table 3:** Optimization of the intramolecular reductive coupling of allene and nitrile to give tetrahydrofuran **19**.

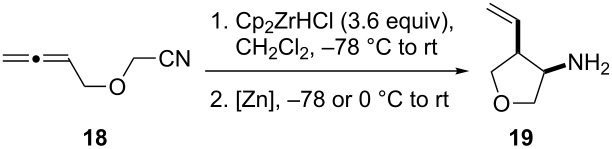			

Entry	Zinc source	Solvent	Yield (%)

1	ZnCl_2_^a^	CH_2_Cl_2_	53
2	ZnCl_2_^b^	CH_2_Cl_2_	50
3	Zn(OTf)_2_^b^	CH_2_Cl_2_	17
4	ZnCl_2_^a^	DCE	55
5	Et_2_Zn^c^	CH_2_Cl_2_	<5
6	Et_2_Zn^d^	CH_2_Cl_2_/Toluene	68
7	Me_2_Zn^d^	CH_2_Cl_2_/Toluene	69

^a^1 M solution in Et_2_O. ^b^Neat salt was added. ^c^1 M solution in CH_2_Cl_2_. ^d^1 M solution in toluene.

Next, we investigated the scope of the intramolecular reaction (Table 4). Both tetrahydrofuran and tetrahydropyran products were obtained in moderate to good yields as single diastereomers, as determined by ^1^H NMR analysis. The conversion of substrate **22** was good, but met with difficulties in isolating the free amine product. Accordingly, treatment of the crude reaction mixture with Boc_2_O and Et_3_N for 2 h at room temperature improved the reaction workup and provided compound **23** in 60% yield.

**Table 4:** Cyclative reductive couplings.^a^

Entry	Substrate	Product	Yield

1	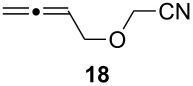	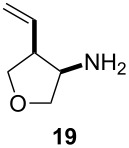	69%^b^
2	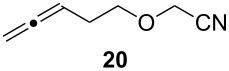	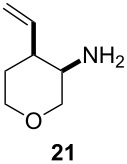	53%^c^
3	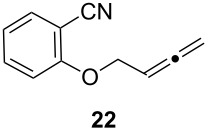	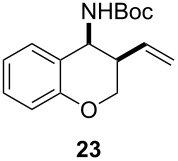	60%^b,d^

^a^All reactions were carried out by hydrozirconation of a mixture of allene (1.4 equiv) and nitrile (1 equiv) in CH_2_Cl_2_ at −78 °C, followed by a solvent switch to toluene and addition of 1 M ZnMe_2_ (entries 1 and 2) or 1 M ZnEt_2_ (entry 3) in toluene (1.4 equiv) at −78 °C. ^b^Relative configuration was assigned in analogy to **21**. ^c^Relative configuration was determined by coupling constant analysis (Figure 1). ^d^Compound **23** was isolated as a Boc-protected amine upon treatment of the crude reaction mixture with Boc_2_O (1 equiv) and Et_3_N (6 equiv) in THF/CH_2_Cl_2_.

**Figure 1:**
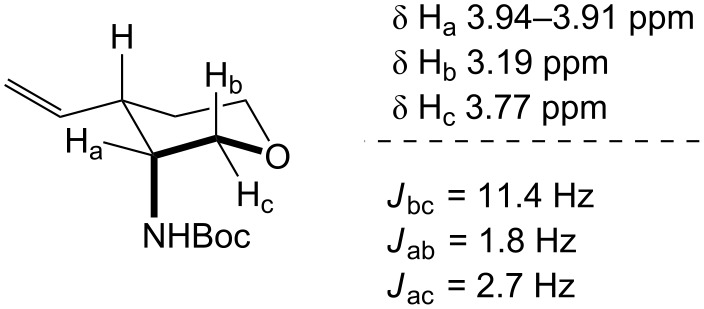
Coupling constant analysis of the Boc-protected aminopyran ring in **21**.

In order to determine the relative configuration of pyran **21**, the amine was protected as the *t*-butyl carbamate (Supporting Information File 1). Signals for both hydrogen atoms H_b_ and H_c_ were doublets of doublets with one large and one small coupling constant. The large coupling constant, *J*_bc_ = 11.4 Hz, corresponds to the geminal coupling between H_b_ and H_c_, while the small coupling constants, *J*_ab_ = 1.8 Hz and *J*_ac_ = 2.7 Hz, correspond to the coupling between H_b/c_ and H_a_. This analysis implies that hydrogen atom H_a_ is in the equatorial position, placing the electronegative carbamate substituent and the C–O bond in the tetrahydropyran ring into a gauche orientation (Figure 1).

**Scheme 3:**
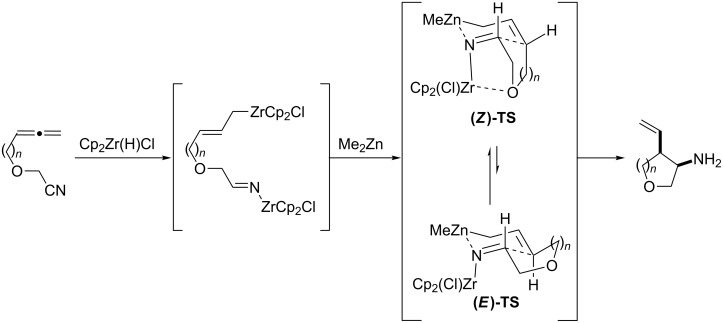
Proposed chelated transition state model.

We propose a chelated transition state for the formation of **19**, **21**, and **23** (Scheme 3). After the initial hydrozirconation and transmetallation with dimethylzinc, both (*E*)- and (*Z*)-allylzinc species can exist in the solution. The chelation of the zirconocene to the ether oxygen and the imine nitrogen leads to a preference for the **(*Z*)-TS** species, paving the way for the formation of the observed *cis*-product.

**Scheme 4:**
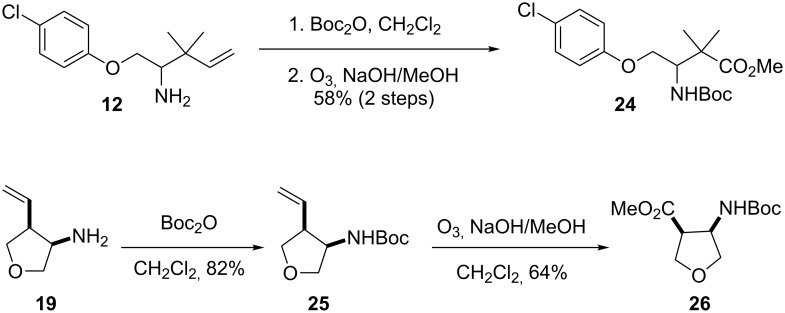
Conversion of homoallylic amines to β-amino acid derivatives.

To further elaborate on the utility of this methodology, we demonstrated that the homoallylic amine products could be readily converted to synthetically useful building blocks, such as β-amino acids (Scheme 4). *N*-Boc-protection of the primary amine **12** followed by ozonolysis under Marshall’s conditions [40] yielded the β-amino acid derivative **24**. The cyclic amine **19** was subjected to analogous reaction conditions to form the tetrahydrofuran β-amino acid derivative **26**.

## Conclusion

We have developed a method for the one-pot simultaneous hydrozirconation of allenes and nitriles to yield allylic zirconocenes and *N*-zirconoimines, respectively. These intermediates can be transmetalated in situ with dimethylzinc or zinc chloride, which facilities the cross-coupling process to give *N*-unprotected homoallylic amines after aqueous workup. All products were isolated as single regio- and diastereoisomers, and the regioselectivity of the allylation step was shown to depend on the allene substitution. The intramolecular variant of this reaction was used to prepare 3-aminotetrahydrofurans and 3-aminotetrahydropyrans, and these addition products can subsequently be transformed into synthetically valuable β-amino acid building blocks.

## Supporting Information

File 1 Experimental procedures and characterization details of synthesized compounds.
