# Supplementary material for: Homoallylic amines by reductive inter- and intramolecular coupling of allenes and nitriles
Source: Beilstein J Org Chem. 2011 Jun 17;7:824–30. doi: 10.3762/bjoc.7.94 (PMC3135093; doi:10.3762/bjoc.7.94)

# Supporting Information

for

# Homoallylic amines by reductive inter- and intramolecular coupling of allenes and nitriles

Peter Wipf* and Marija D. Manojlovic

Address: Department of Chemistry, University of Pittsburgh, 219 Parkman Avenue, Pittsburgh, PA 15260

Email: Peter Wipf* - pwipf@pitt.edu

* Corresponding author

# Experimental procedures and characterization details of synthesized compounds

**General:** All reactions were performed under a nitrogen atmosphere and all glassware was flame dried prior to use. Reactions carried out at −78 °C employed a dry ice/acetone bath. THF was distilled over sodium / benzophenone ketyl, $Et_3N$ and acetonitrile were distilled from $CaH_2$, and $CH_2Cl_2$ and toluene were purified using an alumina filtration system. Compounds **2**, **5**, **7**, **9**, **11**, and **14** were purchased from Aldrich. Schwartz's reagent [1], 2,3-butadien-1-ol [2] and allenes **13** [3] and **16** [4] were prepared according to literature procedures.

Reactions were monitored by TLC analysis (EM Science pre-coated silica gel 60 $F_{254}$ plates, 250 μm layer thickness) and visualization was accomplished with a 254 nm UV light and by staining with a PMA solution (5 g of phosphomolybdic acid in 100 mL of 95% EtOH), *p*-anisaldehyde solution (2.5 mL of *p*-anisaldehyde, 2 mL of AcOH, and 3.5 mL of conc. $H_2SO_4$ in 100 mL of 95% EtOH), Vaughn's reagent (4.8 g of $(NH_4)_6Mo_7O_{24}\cdot 4H_2O$ and 0.2 g of $Ce(SO_4)_2$ in 100 mL of a 3.5 M $H_2SO_4$ solution) or with a $KMnO_4$ solution (1.5 g of $KMnO_4$ and 1.5 g of $K_2CO_3$ in 100 mL of a 0.1% NaOH solution). Flash chromatography on $SiO_2$ was used to purify the crude mixtures.

Melting points were determined on a Mel-Temp II instrument and are reported uncorrected. $^1H$ and $^{13}C$ NMR were obtained on a Bruker Avance 300 instrument. Chemical shifts were reported in parts per million with the residual solvent peak used as an internal standard. $^1H$ NMR spectra were measured at 300 MHz in $CDCl_3$ and tabulated as follows: Chemical shift, multiplicity (s = singlet, d = doublet, t = triplet, q = quartet, dd = doublet of doublet, dt = doublet of triplet, quint = quintet, m = multiplet, b = broad, app = apparent), number of protons, and

coupling constant(s). $^{13}$C NMR spectra were obtained using a proton-decoupled pulse sequence with a d1 of 3 sec, and were tabulated by observed peak. LC/MS analyses were obtained from a Hewlett Packard Series 1100 MSD. Mass spectra were obtained on a Micromass Autospec double focusing instrument. Infrared spectra were measured on a Nicolet AVATAR 360 FT-IR E.S.P. spectrometer (KBr or neat) or Smiths Detection Identify IR FT-IR spectrometer (ATR).

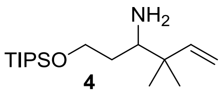

**4**

**4,4-Dimethyl-1-(triisopropylsilyloxy)hex-5-en-3-amine (4). General protocol A.** A flame-dried round bottom flask was charged with nitrile **3** (60 mg, 0.26 mmol) and dry toluene (2.6 mL). The solution was cooled to −78 °C under a nitrogen atmosphere and DIBAL (1 M in hexanes, 0.26 mL, 0.26 mmol) added slowly. The resulting solution was allowed to warm to room temperature over 1 h to give the *N*-diisobutylaluminum imine **1**.

To a suspension of Schwartz's reagent (92 mg, 0.36 mmol) in methylene chloride (0.4 mL) cooled to −78 °C, was added a solution of 3-methyl-1,2-butadiene (**2**) (0.03 mL, 0.31 mmol) in methylene chloride (0.35 mL). The temperature was gradually raised to 0 °C and the reaction mixture stirred at that temperature for 30 min. The resulting red solution was cooled to −78 °C and a solution of the preformed *N*-diisobutylaluminum imine **1** added via cannula. After stirring for 30 min at −78 °C, the cold bath was removed and ethyl acetate and a saturated solution of Rochelle's salt were added with vigorous stirring until the red mixture turned white. The precipitate was filtered through Florisil/Celite (1:1) and

extracted twice with ethyl acetate. The combined organic layers were washed with brine, dried ($Na_2SO_4$), filtered and concentrated in vacuo. The crude residue was purified by chromatography on $SiO_2$ (hexane:EtOAc, 8:2 to 7:3) to afford 58 mg (76%) of **4** as a yellow oil: IR (ATR) 3286, 2939, 2862, 1461, 1379, 1096, 881 $cm^{-1}$; $^{1}H$ NMR δ 5.80 (dd, 1H, *J* = 17.4, 10.8 Hz), 5.04 (dd, 1H, *J* = 10.5, 1.2 Hz), 5.00 (dd, 1H, *J* = 17.7, 9.0 Hz), 3.84 (t, 2H, *J* = 6.0 Hz), 2.65 (dd, 1H, *J* = 10.2, 1.5 Hz), 1.80 (ddt, 1H, *J* = 13.5, 6.6, 1.5 Hz), 1.31–1.19 (m, 3H), 1.08–1.06 (m, 18H), 0.99 (s, 6H); $^{13}C$ NMR δ 146.5, 112.2, 62.6, 57.2, 41.1, 35.1, 23.1, 22.5, 18.0, 11.9; MS (EI) *m*/*z* 299 ($M^+$, 20), 256 (90), 230 (98), 186 (70), 157 (85), 145 (100), 130 (80), 115 (60), 102 (52), 75 (65), 65 (70), 59 (78); HRMS (EI) *m*/*z* calcd for $C_{17}H_{37}NOSi$ 299.2644, found 299.2632.

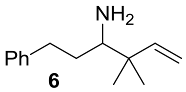

6

**4,4-Dimethyl-1-phenylhex-5-en-3-amine (6).** According to General Protocol A, 3-phenylpropanenitrile **5** (50 mg, 0.37 mmol), DIBAL (1 M in hexanes, 0.37 mL, 0.37 mmol), Schwartz's reagent (133 mg, 0.52 mmol) and 3-methyl-1,2-butadiene **2** (0.03 mL, 0.31 mmol) afforded 52 mg (69%) of **6** as a colorless oil: IR (ATR) 3286, 2958, 2924, 1446, 917, 701 $cm^{-1}$; $^{1}H$ NMR δ 7.32–7.27 (m, 2H), 7.22–7.19 (m, 3H), 5.77 (dd, 1H, *J* = 17.4, 10.8 Hz), 5.04 (dd, 1H, *J* = 10.8, 1.2 Hz), 4.98 (dd, 1H, *J* = 17.4, 1.2 Hz), 2.91 (ddd, 1H, *J* = 13.8, 10.5, 5.1 Hz), 2.57 (ddd, 1H, *J* = 13.5, 9.9, 6.3 Hz), 2.45 (bd, 2H, *J* = 10.5 Hz), 1.96–1.85 (m, 1H), 1.40–1.27 (m, 2H), 0.98 (s, 6H); $^{13}C$ NMR δ 146.4, 142.6, 128.4, 128.3, 125.7, 112.4, 59.1, 41.3, 34.1, 23.2, 22.4; MS (EI) *m*/*z* 204 ($[M + 1]^+$, 40), 160 (25), 135

(75), 117 (80), 98 (47), 91 (100), 77 (42), 69 (58), 65 (70), 56 (52); HRMS (EI) *m*/*z* calcd for $C_{14}H_{22}N$ (M + H) 204.1752, found 204.1758.

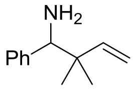


**8**

**2,2-Dimethyl-1-phenylbut-3-en-1-amine (8)** [5]. **General protocol B.** A suspension of Schwartz's reagent (400 mg, 1.55 mmol) in methylene chloride (2.0 mL) was treated at –78 °C under a nitrogen atmosphere with a solution of benzonitrile **7** (44.5 mg, 0.43 mmol) and 3-methyl-1,2-butadiene (**2**) (60 µL, 0.61 mmol) in methylene chloride (0.4 mL). The reaction mixture was slowly warmed to room temperature, stirred for an additional 15 min, cooled to 0 °C, and treated with a solution of $ZnCl_2$ (0.61 mL, 0.61 mmol, 1 M in $Et_2O$). After stirring at room temperature for 3 h, the reaction mixture was poured into a saturated solution of $NaHCO_3$, filtered through celite, extracted with diethyl ether (3×), dried ($Na_2SO_4$) and concentrated. The residue was purified by chromatography on $SiO_2$ (hexanes:EtOAc, 8:2) to yield 56.8 mg (75%) of **8** as a colorless oil. **General protocol C.** A suspension of Schwartz's reagent (434 mg, 1.69 mmol) in methylene chloride (2.1 mL) was treated at −78 °C under a nitrogen atmosphere with a solution of benzonitrile **7** (48.0 mg, 0.47 mmol) and 3-methyl-1,2-butadiene (**2**) (65 µL, 0.66 mmol) in methylene chloride (0.5 mL). The reaction mixture was slowly warmed to room temperature and stirred for an additional 15 min. Methylene chloride was then carefully removed under vacuum until less than 10% of the volume was left in the flask, and toluene (2.6 mL) added. The clear,

dark red solution was cooled to −78 °C, and a solution of $Me_2Zn$ (0.66 mL, 0.66 mmol, 1 M in toluene) added. After stirring at room temperature for 3 h, the reaction mixture was poured into a saturated solution of $NaHCO_3$, filtered through celite, extracted with diethyl ether (3×), dried ($Na_2SO_4$), filtered through a plug of Florisil and concentrated. The residue was purified by chromatography on $SiO_2$ (hexanes:EtOAc, 8:2) to afford 64 mg (78%) of **8** as a colorless oil: IR (ATR) 2691, 2927, 1632, 1450, 917, 719, 701 $cm^{-1}$; $^{1}H$ NMR δ 7.31–7.24 (m, 5H), 5.88 (dd, 1H, *J* = 17.4, 10.8 Hz), 5.11 (dd, 1H, *J* = 10.8, 1.2 Hz), 5.05 (dd, 1H, *J* = 17.4, 1.2 Hz), 3.77 (bs, 1H), 1.56 (bs, 2H), 1.00 (s, 3H), 0.96 (s, 3H); $^{13}C$ NMR δ 145.5, 142.3, 128.4, 127.5, 127.0, 113.2. 64.1, 29.7, 25.4, 21.8.

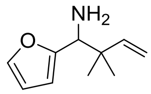

**10**

**1-(Furan-2-yl)-2,2-dimethylbut-3-en-1-amine (10).** According to the general protocol B, furonitrile **9** (47 µL, 0.54 mmol), methyl-1,2-butadiene **2** (75 µL, 0.76 mmol), Schwartz's reagent (500 mg, 1.94 mmol), and $ZnCl_2$ (0.76 mL, 0.76 mmol, 1 M in $Et_2O$) afforded 48.9 mg (55%) of **10** as a pale yellow oil. According to the general protocol C, furonitrile **9** (42 µL, 0.49 mmol), methyl-1,2-butadiene (**2**) (68 µL, 0.68 mmol), Schwartz's reagent (450 mg, 1.75 mmol), and $Me_2Zn$ (0.68 mL, 0.68 mmol, 1 M in toluene) afforded 52.9 mg (67%) of **10** as a pale yellow oil: IR (ATR) 2954, 2920, 2851, 1725, 1271, 1122, 736 $cm^{-1}$; $^{1}H$ NMR δ 7.33 (dd, 1H, *J* = 1.8, 0.9 Hz), 6.32 (dd, 1H, *J* = 3.3, 1.8 Hz), 6.14 (d, 1H, *J* = 3.3 Hz), 5.88 (dd, 1H, *J* = 17.4, 10.8 Hz), 5.10 (dd, 1H, *J* = 10.8, 1.2 Hz), 5.06 (dd, 1H, *J* = 17.4, 1.5

Hz), 3.76 (s, 1H), 1.04 (s, 3H), 1.01 (s, 3H); $^{13}$C NMR δ 145.4, 140.9, 113.1, 109.8, 106.4, 58.3, 36.6, 24.8, 22.2; HRMS (EI) *m*/*z* calcd for $C_{10}H_{15}NO$ 165.1154, found 165.1147.

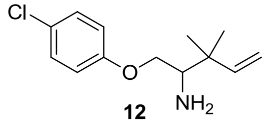

**12**

**1-(4-Chlorophenoxy)-3,3-dimethylpent-4-en-2-amine (12).** According to the general protocol B, nitrile **11** (39 mg, 0.22 mmol), methyl-1,2-butadiene (**2**) (31 μL, 0.31 mmol), Schwartz's reagent (0.21 g, 0.80 mmol), and $ZnCl_2$ (0.31 mL, 0.31 mmol, 1 M in $Et_2O$) afforded 42 mg (80%) of **12** as a colorless oil. According to the general protocol C, nitrile **11** (37 mg, 0.21 mmol), methyl-1,2-butadiene (**2**) (30 μL, 0.30 mmol), Schwartz's reagent (0.20 g, 0.76 mmol), and $Me_2Zn$ (0.29 mL, 0.29 mmol, 1 M in toluene) afforded 40.8 mg (81%) of **12** as a colorless, slightly impure oil: IR (ATR) 3397, 2961, 2870, 1591, 1489, 1239, 812 cm$^{-1}$; $^{1}$H NMR δ 7.22 (d, 2H, *J* = 9.0 Hz), 6.83 (d, 2H, *J* = 9.0 Hz), 5.88 (dd, 1H, *J* = 17.4, 10.8 Hz), 5.09 (dd. 1H, *J* = 10.8, 1.2 Hz), 5.07 (dd, 1H, *J* = 17.4, 1.2 Hz), 4.07 (dd, 1H, *J* = 9.3, 3.0 Hz), 3.68 (t, 1H, *J* = 8.7 Hz), 3.00 (dd, 1H, *J* = 8.7, 2.7 Hz), 1.67 (bs, 2H), 1.11 (s, 6H); $^{13}$C NMR δ 157.6, 145.3, 129.3, 125.8, 116.0, 112.9, 70.6, 58.1, 39.7, 23.6, 23.2; MS (EI) *m*/*z* 239 ($M^+$, 21), 214 (20), 172 (22), 170 (65), 156 (10), 153 (12), 84 (10), 82 (15), 69 (22), 57 (100), 55 (15); HRMS (EI) *m*/*z* calcd for $C_{13}H_{18}NOCl$ 239.1077, found 239.1076.

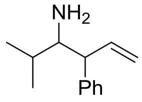


**15**

**2-Methyl-4-phenylhex-5-en-3-amine (15).** According to general protocol B, nitrile **14** (28 mg, 0.39 mmol), allene **13** (65 mg, 0.55 mmol), Schwartz's reagent (365 mg, 1.42 mmol), and $ZnCl_2$ (0.55 mL, 0.55 mmol, 1 M in $Et_2O$) afforded 48 mg (65%) of **15** as a colorless oil. According to the general protocol C, nitrile **14** (30 mg, 0.42 mmol), allene **13** (70 mg, 0.59 mmol), Schwartz's reagent (391 mg, 1.52 mmol), and $ZnMe_2$ (0.59 mL, 0.59 mmol, 1 M in toluene) afforded 57 mg (71%) of **15** as a colorless, slightly EtOAc-contaminated oil: IR (ATR) 2956, 2922, 2868, 1634, 1491, 1451, 917, 701 $cm^{-1}$; $^{1}H$ NMR δ 7.31–7.29 (m, 2H), 7.24–7.18 (m, 3H), 6.06 (ddd, 1H, *J* = 16.8, 9.9, 9.6 Hz), 5.19–5.12 (m, 2H), 3.21 (app t, 1H, *J* = 9.0 Hz), 2.88 (dd, 1H, *J* = 8.7, 3.9 Hz), 1.55–1.46 (m, 2H), 0.93 (d, 3H, *J* = 6.9 Hz), 0.82 (d, 3H, *J* = 6.6 Hz); $^{13}C$ NMR δ 142.9, 140.0, 128.6, 127.7, 126.3, 116.6, 59.6, 55.8, 28.9, 20.9, 14.9; HRMS (EI) *m*/*z* calcd for $C_{13}H_{19}N$ 189.1517, found 189. 1525.

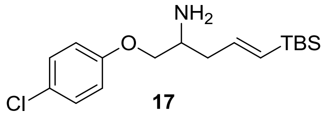


**17**

**(*E*)-5-(*tert*-Butyldimethylsilyl)-1-(4-chlorophenoxy)pent-4-en-2-amine (17).** According to general protocol B, nitrile **11** (50 mg, 0.30 mmol), allene **16** (64 mg, 0.42 mmol), Schwartz's reagent (277 mg, 1.07 mmol), and $ZnCl_2$ (0.42 mL, 0.42 mmol, 1 M in $Et_2O$) afforded 65 mg (67%) of **17** as a colorless oil. According to the general protocol C, nitrile **11** (50 mg, 0.30 mmol), allene **16** (64 mg, 0.42

mmol), Schwartz's reagent (277 mg, 1.07 mmol), and $ZnMe_2$ (0.42 mL, 0.42 mmol, 1 M in toluene) afforded 68 mg (70%) of **17** as a colorless oil: IR (ATR) 3286, 2948, 2924, 2853, 1591, 1489, 1241, 822 $cm^{-1}$; $^1$H NMR δ 7.23 (d, 2H, *J* = 9.0 Hz), 6.83 (d, 2H, *J* = 9.0 Hz), 6.04 (dt, 1H, *J* = 18.6, 6.9 Hz), 5.79 (app d, 1H, *J* = 18.6 Hz), 3.90 (dd, 1H, *J* = 9.0, 4.2 Hz), 3.74 (dd, 1H, *J* = 8.7, 7.2 Hz), 3.28 (app bs, 1H), 2.42 (dt, 1H, *J* = 13.8, 5.1 Hz), 2.25 (dt, 1H, *J* = 13.8, 6.9 Hz), 0.87 (s, 9H), 0.03 (s, 6H); $^{13}$C NMR δ 157.5, 143.8, 131.6, 129.3, 125.7, 115.8, 73.0, 49.9, 41.9, 26.4, 16.4, −6.1; MS (CI) *m/z* 326 ($[M + 1]^+$, 100), 172 (40), 170 (58), 114 (38), 102 (22); HRMS (EI) *m/z* calcd for $C_{17}H_{28}NOSiCl$ 325.1629, found 325.1631.

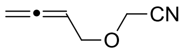


**18**

**2-(Buta-2,3-dienyloxy)acetonitrile (18).** A mixture of 2,3-butadien-1-ol (1.3 g, 18 mmol) and NaH (2.3 g, 93 mmol) in dry acetonitrile (23.5 mL) was stirred at room temperature for 1 h and then bromoacetonitrile (7.1 mL, 102 mmol) was added at −78 °C. The reaction mixture was gradually warmed to room temperature and stirred for 18 h and the acetonitrile was removed under reduced pressure. The black viscous residue was suspended in $CH_2Cl_2$ and the suspension filtered through Celite. Upon concentration, the crude mixture was purified by chromatography on $SiO_2$ (pentane:$Et_2O$, 9:1) followed by the removal of bromoacetonitrile at 25 mmHg to afford 1.1 g of **18** (54%): IR (ATR) 2923, 2360, 2340, 1955, 1436, 1355, 1097, 981, 853 $cm^{-1}$; $^1$H NMR δ 5.22 (app quint, 1H, *J* = 6.9 Hz), 4.89 (dt, 2H, *J* = 6.6, 2.1 Hz), 4.28 (s, 2H), 4.17 (dt, 2H, *J* = 6.9,

2.1 Hz); $^{13}C$ NMR δ 210.2, 115.8, 85.8, 76.4, 69.0, 54.4; HRMS (EI) *m/z* calcd for $C_6H_7NO$ 109.0528, found 109.0521.

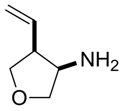

**19**

**(3*R**,4*S**)-4-Vinyltetrahydrofuran-3-amine (19).** A suspension of Schwartz's reagent (298 mg, 1.16 mmol) in methylene chloride (1.2 mL) was treated at −78 °C under a nitrogen atmosphere with a solution of **18** (35 mg, 0.32 mmol) in methylene chloride (0.6 mL). The reaction mixture was slowly warmed to room temperature and stirred for an additional 15 min. Methylene chloride was then carefully removed under vacuum until less than 10% of the volume was left in the flask, and toluene (1.8 mL) was added. The clear, dark red solution was cooled to −78 °C and a solution of $Me_2Zn$ (0.45 mL, 0.45 mmol, 1 M in toluene) added. After stirring at room temperature for 3h, the reaction mixture was poured into a saturated solution of $NaHCO_3$, filtered through celite, extracted with diethyl ether (3×), dried ($Na_2SO_4$), filtered through a plug of Florisil and concentrated. The residue was purified by chromatography on $SiO_2$ ($CH_2Cl_2$:MeOH, 95:5) to afford 25 mg (69%) of **19** (relative configuration was tentatively assigned as *cis* based on the analogy with compound **21**) as a colorless oil: IR (neat) 3364, 3286, 2926, 2862, 1647, 1591, 1575, 1075, 917 $cm^{-1}$; $^1H$ NMR δ 5.85 (ddd, 1H, *J* = 17.4, 10.5, 8.1 Hz), 5.24 (ddd, 1H, *J* = 10.2, 1.5, 0.6 Hz), 5.18 (ddd, 1H, *J* = 17.4, 1.8, 0.9 Hz), 4.02–3.91 (m, 1H), 3.96 (dd, 1H, *J* = 8.4, 7.5 Hz), 3.77 (app t, 1H, *J* = 8.4 Hz), 3.71–3.47 (m, 1H), 3.58 (dd, 1H, *J* = 8.7, 3.3 Hz), 2.87 (app quint, 1H, *J*

= 6.6 Hz); $^{13}$C NMR δ 136.8, 116.9, 74.7, 72.1, 57.9, 54.1; MS (CI) 114 ([M + 1]$^+$, 100), 100 (75).

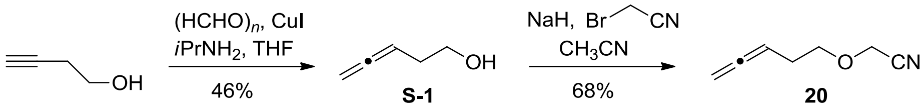


**Penta-3,4-dien-1-ol (S-1)** [6]**.** To a suspension of 3-butyn-1-ol (2.0 g, 28 mmol), diisopropylamine (8.0 mL, 57 mmol) and paraformaldehyde (2.1 g, 71 mmol) in THF (55 mL), was added copper(I) iodide (1.4 g, 7 mmol) in small portions with vigorous stirring. The reaction mixture was heated under reflux for 16 h, filtered through celite and concentrated to a thick brown oil. The residue was diluted with water (15 mL) and ether (20 ml) and acidified to pH 3 with 3 M HCl. The suspension was filtered, the aqueous layer extracted with ether (4×), and the organic layers were washed successively with water and brine, dried ($Na_2SO_4$) and concentrated. The residue was purified by Kugelrohr distillation (60 °C, 20 Torr) to afford 1.1 g (46%) of **S-1** as a colorless liquid: IR (neat) 3336, 2943, 1955, 1428, 1046, 840 cm$^{-1}$; $^1$H NMR δ 5.13 (app quint, 1H, *J* = 6.9 Hz), 4.74 (dt, 2H, *J* = 6.6, 3.0 Hz), 3.74–3.71 (m, 2H), 2.28 (app dquint, 2H, *J* = 6.3, 3.0 Hz), 1.69 (bs, 1H); $^{13}$C NMR δ 209.0, 86.4, 75.2, 61.9, 31.6; MS (EI) *m*/*z* 84 (M$^+$, 25), 69 (100), 66 (22), 53 (78); HRMS (EI) *m*/*z* calcd for $C_5H_8O$ 84.0575, found 84.0575.

**2-(Penta-3,4-dienyloxy)acetonitrile (20).** A mixture of penta-3,4-dien-1-ol **S-1** (0.80 g, 9.5 mmol) and NaH (1.1 g, 47.6 mmol) in acetonitrile (12.5 mL) was

stirred at room temperature for 1 h and bromoacetonitrile (3.6 mL, 52.3 mmol) added dropwise at −40 °C. The reaction mixture was gradually warmed to room temperature and stirred for 14 h. Acetonitrile was removed under reduced pressure, and the residue diluted with $CH_2Cl_2$ and filtered through celite. Upon concentration, the mixture was purified by chromatography on $SiO_2$ (pentane:ether, 9:1) to afford 0.81 g of **20** (68%) as a colorless liquid: IR (neat) 3021, 2920, 2253, 1955, 1413, 1211, 1107, 941, 857 $cm^{-1}$; $^{1}H$ NMR δ 5.13 (app quint, 1H, *J* = 6.9 Hz), 4.74 (dt, 2H, *J* = 6.6, 3.3 Hz), 4.27 (s, 2H), 3.67 (t, 2H, *J* = 6.6 Hz), 2.34 (dtt, 2H, *J* = 6.6, 6.3, 3.0 Hz); $^{13}C$ NMR δ 208.9, 116.0, 85.9, 75.6, 70.8, 56.2, 28.1; HRMS (EI) *m/z* calcd for $C_7H_9O$ 123.0684, found 123.0678.

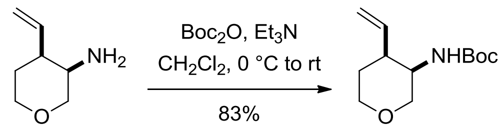


**21** **21a**

***tert*-Butyl (3*R**,4*S**)-4-vinyltetrahydro-2H-pyran-3-ylcarbamate (21a).** A suspension of Schwartz's reagent (339 mg, 1.32 mmol) in methylene chloride (1.5 mL) was treated at −78 °C under a nitrogen atmosphere with the solution of **20** (45 mg, 0.37 mmol) in methylene chloride (0.7 mL). The reaction mixture was slowly warmed to room temperature and stirred for an additional 15 min. Methylene chloride was then carefully removed under vacuum until less than 10% of the volume was left in the flask and toluene (2.2 mL) added. The clear, dark red solution was cooled to −78 °C and a solution of $Me_2Zn$ (0.51 mL, 0.51 mmol, 1 M in toluene) added. After stirring at room temperature for 3 h, the reaction mixture was poured into a saturated solution of $NaHCO_3$, filtered through

celite, extracted with diethyl ether (3×), dried ($Na_2SO_4$), filtered through a plug of Florisil and concentrated to afford 25 mg (53%) of crude **21**. The crude product **21** was immediately dissolved in $CH_2Cl_2$ (0.40 mL) and $Et_3N$ (30 μL, 0.22 mmol) followed by the addition of a solution of di-*tert*-butyl dicarbonate (43 mg, 0.20 mmol) in $CH_2Cl_2$ (0.2 mL) at 0 °C. Upon stirring for 2 h at room temperature, a saturated solution of $NH_4Cl$ was added and the mixture extracted with EtOAc. The organic layer was washed with brine, dried ($Na_2SO_4$), and concentrated in vacuo. The residue was purified by chromatography on $SiO_2$ (hexane:EtOAc, 1:1) to afford 37 mg (<44%, 2 steps) of slightly impure **21a** as a colorless oil: IR (ATR) 3343, 2971, 2930, 2846, 1705, 1495, 1364, 1163, 1099, 997 $cm^{-1}$; $^1H$ NMR δ ($CDCl_3$) 5.86 (ddd, 1H, *J* = 17.1, 10.8, 6.3 Hz), 5.08 (dt, 1H, *J* = 10.5, 1.2 Hz), 5.05 (app d, 1H, *J* = 17.1 Hz), 4.98 (bd, 1H, *J* = 8.1 Hz), 3.95 (app dt, 1H, *J* = 11.1, 3.3 Hz), 3.80 (d, 2H, *J* = 10.2 Hz), 3.53 (d, 1H, *J* = 9.6 Hz), 3.46 (dt, 1H, *J* = 11.1, 3.3 Hz), 2.50–2.40 (bm, 1H), 1.71–1.54 (m, 2H), 1.42 (s, 9H); $^1H$ NMR δ ($C_6D_6$) 5.95 (ddd, 1H, *J* = 17.1, 10.5, 6.0 Hz), 5.09 (dt, 1H, *J* = 10.5, 1.2 Hz), 5.04 (bs, 1H), 4.96 (dt, 1H, *J* = 17.4, 1.8 Hz), 3.94–3.91 (bm, 1H), 3.77 (dd, 1H, *J* = 11.4, 2.7 Hz), 3.67 (dt, 1H, *J* = 11.4, 3.9 Hz), 3.19 (dd, 1H, *J* = 11.4, 1.8 Hz), 3.06 (dt, 1H, *J* = 11.4, 2.7 Hz), 2.08–2.00 (m, 1H), 1.56 (s, 9H), 1.30–1.19 (m, 1H), 1.14–1.08 (m, 1 H); $^{13}C$ NMR δ ($CDCl_3$) 155.5, 138.6, 115.2, 79.1, 71.4, 67.2, 48.6, 41.3, 28.3, 26.4; MS (EI) *m*/*z* 227 ($M^+$, 18), 121 (30), 119 (95), 117 (100), 86 (50), 84 (80), 82 (25), 57 (11); HRMS (EI) *m*/*z* calcd for $C_{12}H_{21}NO_3$ 227.1521, found 227.1520.

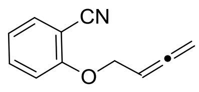

**22**

**2-(Buta-2,3-dienyloxy)benzonitrile (22).** To a solution of 2-cyanophenol (500 mg, 4.20 mmol), 2,3-butadien-1-ol (354 mg, 5.05 mmol) and $Ph_3P$ (1.32 g, 5.05 mmol) in THF (8.8 mL) at 0 °C, was added DIAD (1.0 mL, 5.1 mmol) dropwise. The reaction mixture was stirred at 0 °C for 30 min and at room temperature for 3 h. Upon concentration under reduced pressure, the crude reaction mixture was purified by chromatography on $SiO_2$ (hexane:EtOAc, 9:1) to afford 568 mg (79%) of **22** as a colorless oil: IR (ATR) 3086, 3037, 2225, 1955, 1724, 1595, 1487, 1448, 1286, 1252, 1226, 992, 848, 751 $cm^{-1}$; $^1H$ NMR δ 7.52–7.48 (m, 2H), 7.02–6.96 (m, 2H), 5.37 (app quint, 1H, *J* = 6.6 Hz), 4.87 (dt, 2H, *J* = 6.6, 2.4 Hz), 4.68 (dt, 2H, *J* = 6.6, 2.4 Hz); $^{13}C$ NMR δ 209.4, 159.8, 134.2, 133.5, 120.4, 116.3, 112.0, 102.1, 86.1, 76.9, 64.4; MS (APCI) *m*/*z* 173 ($[M + 1]^+$, 25), 172 ($M^+$, 100), 155 (15); HRMS (APCI) *m*/*z* calcd for $C_{11}H_{10}NO$ 172.0762, found 172.0779.

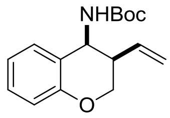

**23**

***tert*-Butyl (3*R**,4*S**)-3-vinylchroman-4-ylcarbamate (23).** A suspension of Schwartz's reagent (541 mg, 2.10 mmol) in methylene chloride (2.6 mL) was treated at −78 °C under a nitrogen atmosphere with the solution of **22** (0.10 g, 0.58 mmol) in methylene chloride (0.6 mL). The reaction mixture was slowly warmed to room temperature and stirred for an additional 15 min. Methylene chloride was then carefully removed under vacuum until less than 10% of the

volume was left in the flask and toluene (3.2 mL) added. The clear, dark red solution was cooled to −78 °C and $Et_2Zn$ (0.82 mL, 0.82 mmol, 1 M in toluene) added. After stirring at room temperature for 3 h, the reaction mixture was poured into concentrated $NH_4OH$ solution, filtered through Celite, extracted with diethyl ether (3×), dried ($Na_2SO_4$), filtered through a plug of florisil and concentrated. The crude amine was immediately dissolved in THF (4.3 mL) and $Et_3N$ (0.49 mL, 3.5 mmol) followed by addition of a solution of di-*tert*-butyl dicarbonate (0.13 g, 0.58 mmol) in $CH_2Cl_2$ (1.5 mL) at 0 °C. Upon stirring for 2 h at room temperature, water was added and the mixture extracted with EtOAc. The combined organic layers were washed with brine, dried ($Na_2SO_4$), and concentrated in vacuo. The residue was purified by chromatography on $SiO_2$ (hexane:EtOAc, 9:1) to afford 71 mg (60%, 2 steps) of **23** as a white solid: Mp 87–88 °C; IR (ATR) 3314, 2977, 2928, 1676, 1515, 1487, 1454, 1221, 1167, 1072, 1049, 917, 755 $cm^{-1}$; $^1H$ NMR δ 7.27 (d, 1H, *J* = 7.5 Hz), 7.18 (dt, 1H, *J* = 8.1, 1.5 Hz), 6.92 (dt, 1H, *J* = 7.5, 0.9 Hz), 6.82 (d, 1H, *J* = 8.1 Hz), 5.85 (ddd, 1H, *J* = 18, 9.6, 8.7 Hz), 5.29–5.24 (m, 2H), 5.02 (dd, 1H, *J* = 9.3, 5.4 Hz), 4.73 (d, 1H, *J* = 9.0 Hz), 4.31 (dd, 1H, *J* = 11.1, 2.4 Hz), 4.12 (dd, 1H, *J* = 11.4, 6.3 Hz), 2.87–2.85 (m, 1H), 1.49 (s, 9H); $^{13}C$ NMR δ 155.7, 154.2, 133.5, 129.0, 128.7, 122.3, 120.9, 119.1, 79.7, 67.6, 47.8, 41.0, 28.4.

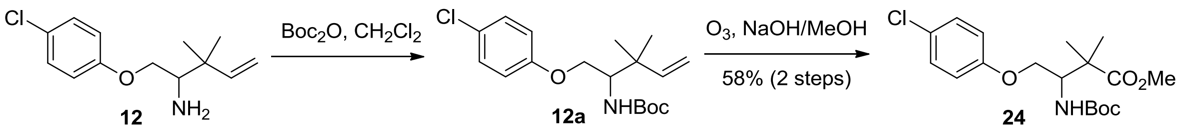

**tert-Butyl 1-(4-chlorophenoxy)-3,3-dimethylpent-4-en-2-ylcarbamate (12a).** To a solution of **12** (115 mg, 0.480 mmol) in $CH_2Cl_2$ (0.65 mL) at 0 °C, was added a solution of di-*tert*-butyl dicarbonate (108 mg, 0.480 mmol) in $CH_2Cl_2$ (0.85 mL) dropwise over 20 min. The reaction mixture was stirred at 0 °C for 2 h, the reaction quenched with water and extracted with ethyl acetate. The combined organic layers were washed with brine and dried ($Na_2SO_4$). Concentration in vacuo afforded crude **12a** as a white solid that was used without further purification: Mp 100.5–101 °C; IR (neat) 3317, 2965, 1677, 1539, 1491, 1461, 1241, 1167 $cm^{-1}$; $^1$H NMR δ 7.23 (dt, 2H, *J* = 9.0, 3.3 Hz), 6.80 (d, 2H, *J* = 8.7 Hz), 5.92 (dd, 1H, *J* = 17.4, 10.8 Hz), 5.09 (d, 1H, 10.2 Hz), 5.06 (d, 1H, *J* = 17.4 Hz), 4.77 (d, 1H, *J* = 9.6 Hz), 4.04 (dd, 1H, *J* = 9.6, 3.6 Hz), 3.93–3.86 (m, 2H), 1.45 (s, 9H), 1.21 (s, 3H), 1.10 (s, 3 H); MS (EI) *m*/*z* 239 ($[M - Boc]^+$, 22), 214 (17), 172 (20), 170 (65), 153 (12), 82 (13), 69 (22), 57 (100), 55 (15).

**Methyl 3-(*tert*-butoxycarbonylamino)-4-(4-chlorophenoxy)-2,2-dimethyl-butanoate (24).** A solution of **12a** (80 mg, 0.24 mmol) in $CH_2Cl_2$ (2 mL) and 2.5 M NaOH in methanol (0.50 mL) was stirred at −78 °C and ozone bubbled through the initially orange-yellow reaction mixture until it acquired a blue color. The reaction mixture was diluted with ether and water, allowed to warm to room temperature and extracted with ether. The organic layer was dried ($MgSO_4$) and concentrated in vacuo. The residue was purified by chromatography on $SiO_2$

(hexane:EtOAc, 1:1) to afford 90 mg (58%, 2 steps) of **24** as a colorless oil: IR (ATR) 3440, 2974, 2926, 1705, 1489, 1364, 1159, 822 $cm^{-1}$; $^1H$ NMR δ 7.26 (d, 2H, *J* = 9.3 Hz), 6.77 (d, 2H, *J* = 9.3 Hz), 5.51 (bd, 1H, *J* = 9.6 Hz), 5.12–5.04 (m, 1H), 4.08–4.03 (m, 2H), 3.71 (s, 3H), 1.46 (s, 9H), 1.30 (s, 6 H); $^{13}C$ NMR δ 176.8, 157.0, 155.8, 129.4, 129.3, 126.0, 115.8, 79.6, 68.2, 56.3, 52.0, 44.4, 28.4, 23.7, 23.4; MS (EI) *m*/*z* 371 ($M^+$, 17), 298 (25), 284 (17), 230 (33), 195 (37), 188 (100), 174 (70), 130 (72), 128 (45), 85 (60); HRMS (EI) *m*/*z* calcd for $C_{18}H_{26}NO_5Cl$ 371.1500, found 371.1499.

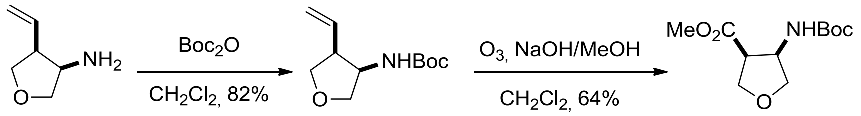


***tert*-Butyl (3*R**,4*S**)-4-vinyltetrahydrofuran-3-ylcarbamate (25).** A solution of amine **19** (85 mg, 0.75 mmol) in $CH_2Cl_2$ (1.3 mL) and $Et_3N$ (0.12 mL, 0.83 mmol) was treated with a solution of di-*tert*-butyl dicarbonate (161 mg, 0.751 mmol) in $CH_2Cl_2$ (0.7 mL) at 0 °C. After stirring for 2 h at room temperature, a saturated solution of $NH_4Cl$ was added and the mixture extracted with EtOAc. The organic layer was washed with brine, dried ($Na_2SO_4$) and concentrated in vacuo. The residue was purified by chromatography on $SiO_2$ (pentane:ether, 1:1) to afford 131 mg (82%) of **25** as a colorless, thick oil: IR (ATR) 3315, 2972, 2931, 1696, 1524, 1364, 1249, 1174 $cm^{-1}$; $^1H$ NMR δ 5.78 (ddd, 1H, *J* = 17.4, 9.9, 7.5 Hz), 5.18 (dt, 1H, 17.4, 1.2 Hz), 5.13 (ddd, 1H, *J* = 10.2, 1.2, 0.9 Hz), 4.65 (m, 1H), 4.31 (bm, 1H), 4.02 (dd, 1H, *J* = 9.3, 5.7 Hz), 3.98 (dd, 1H, *J* = 8.7, 7.5 Hz), 3.72 (dd, 1H, *J* = 8.7, 7.2 Hz), 3.62 (dd, 1H, *J* = 9.0, 4.2 Hz), 3.00 (app quint, 1H, *J* =

6.9 Hz), 1.44 (s, 9 H); $^{13}$C NMR δ 155.4, 133.7, 118.7, 79.6, 73.2, 70.8, 53.7, 46.2, 28.3; MS (CI) *m*/*z* 213 ($M^+$, 4), 177 (2), 158 (2).

**(3*S**,4*R**)-Methyl 4-(*tert*-butoxycarbonylamino)tetrahydrofuran-3-carboxylate (26).** A solution of **25** (51 mg, 0.24 mmol) in $CH_2Cl_2$ (2 mL) and 2.5 M NaOH in methanol (0.50 mL) was stirred at −78 °C and ozone was bubbled through the initially orange-yellow reaction mixture until it acquired a blue color. The reaction mixture was diluted with ether and water, allowed to warm to room temperature and extracted with ether. The combined organic layers were dried ($MgSO_4$) and concentrated in vacuo. The residue was purified by chromatography on $SiO_2$ (hexane:EtOAc, 1:1) to afford 38 mg (64%) of **26** as a colorless oil: IR (ATR) 3268, 2972, 2902, 1709, 1518, 1364, 1159, 1068 cm$^{-1}$; $^{1}$H NMR δ 5.09 (bd, 1H, *J* = 8.1 Hz), 4.58 (bm, 1H), 4.11 (dd, 1H, *J* = 9.0, 7.2 Hz), 4.03 (app t, 1H, *J* = 8.1 Hz), 3.94 (dd, 1H, *J* = 9.0, 5.7 Hz), 3.72 (s, 3H), 3.67 (dd, 1H, *J* = 9.0, 4.5 Hz), 3.32 (app q, 1H, *J* = 7.5 Hz), 1.43 (s, 9H); $^{13}$C NMR δ 171.8, 155.2, 79.9, 73.0, 69.3, 53.0, 52.1, 47.5, 28.3; MS (EI) *m*/*z* 245 ($M^+$, 12), 230 (8), 189 (47), 172 (52), 158 (45), 140 (45), 128 (80), 114 (37), 87 (85), 69 (100); HRMS (EI) *m*/*z* calcd for $C_{11}H_{19}NO_5$ 245.1263, found 245.1259.

## References


1. Buchwald, S. L.; La Maire, S. J.; Nielsen, R. B. *Org. Synth.* **1993,** *71,* 77–80. http://www.orgsyn.org/orgsyn/pdfs/CV9P0162.pdf.

2. Bennacer, B.; Fujiwara, M.; Lee, S.-Y.; Ojima, I. *J. Am. Chem. Soc.* **2005,** *127,* 17756–17767. doi:10.1021/ja054221m

3. Myers, A. G.; Zheng, B. J. *J. Am. Chem. Soc.* **1996,** *118,* 4492–4493. doi:10.1021/ja960443w

4. Brandsma, L.; Verkruijsse, H. D. In *Synthesis of Acetylenes, Allenes and Cumulenes*; Elsevier: Amsterdam, 1981; pp 157–160.

5. Manabe, S. *Tetrahedron Lett.* **1997,** *38,* 2491–2492. doi:10.1016/S0040-4039(97)00374-2

6. Price, W.; Patten, T. *J. Chem. Educ.* **1991,** *68,* 256–257. doi:10.1021/ed068p256

mdm-II-063 1/8/08 301 NMR CDCl3

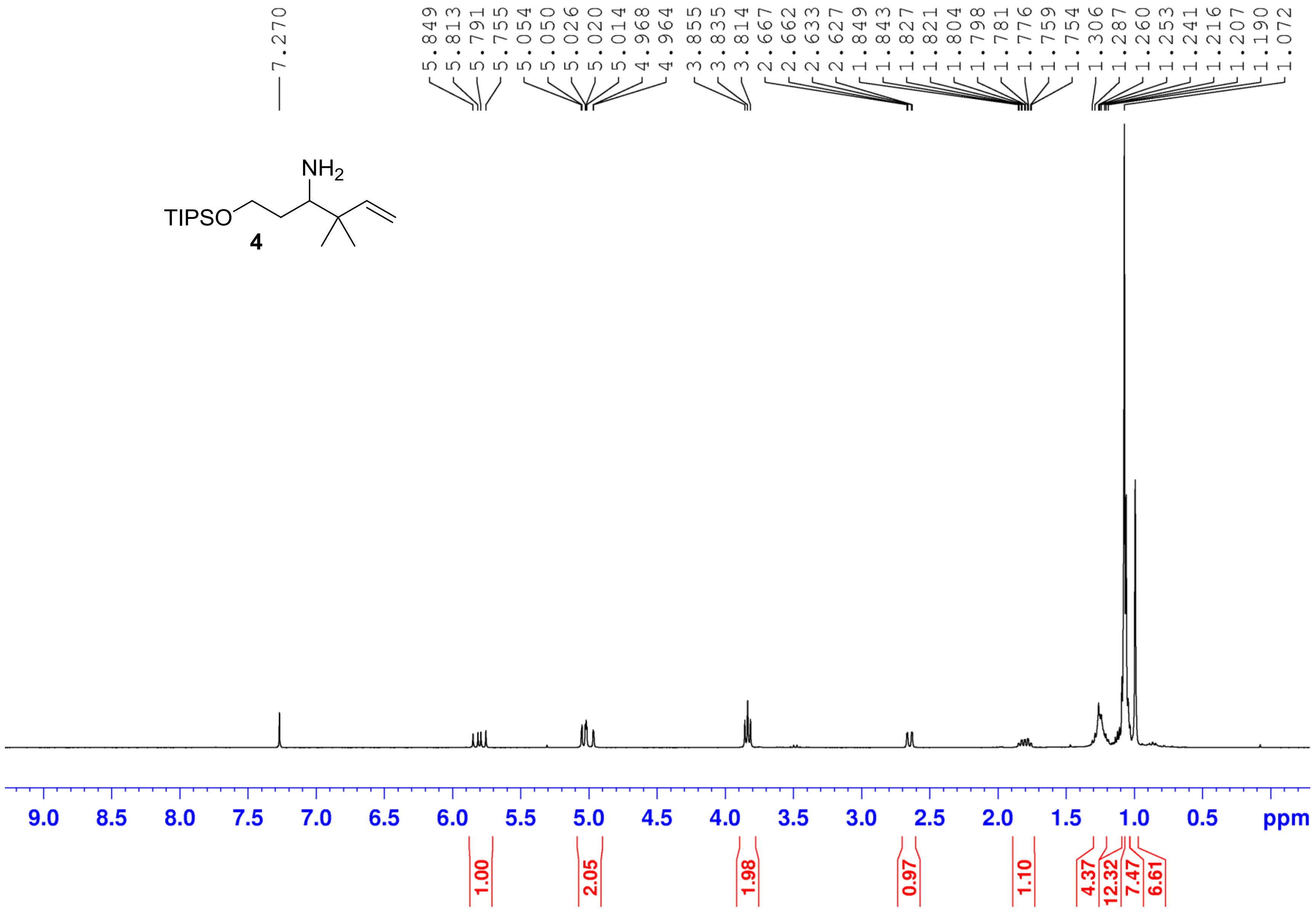

mdm-II-063 1/8/08 301 NMR CDCl3

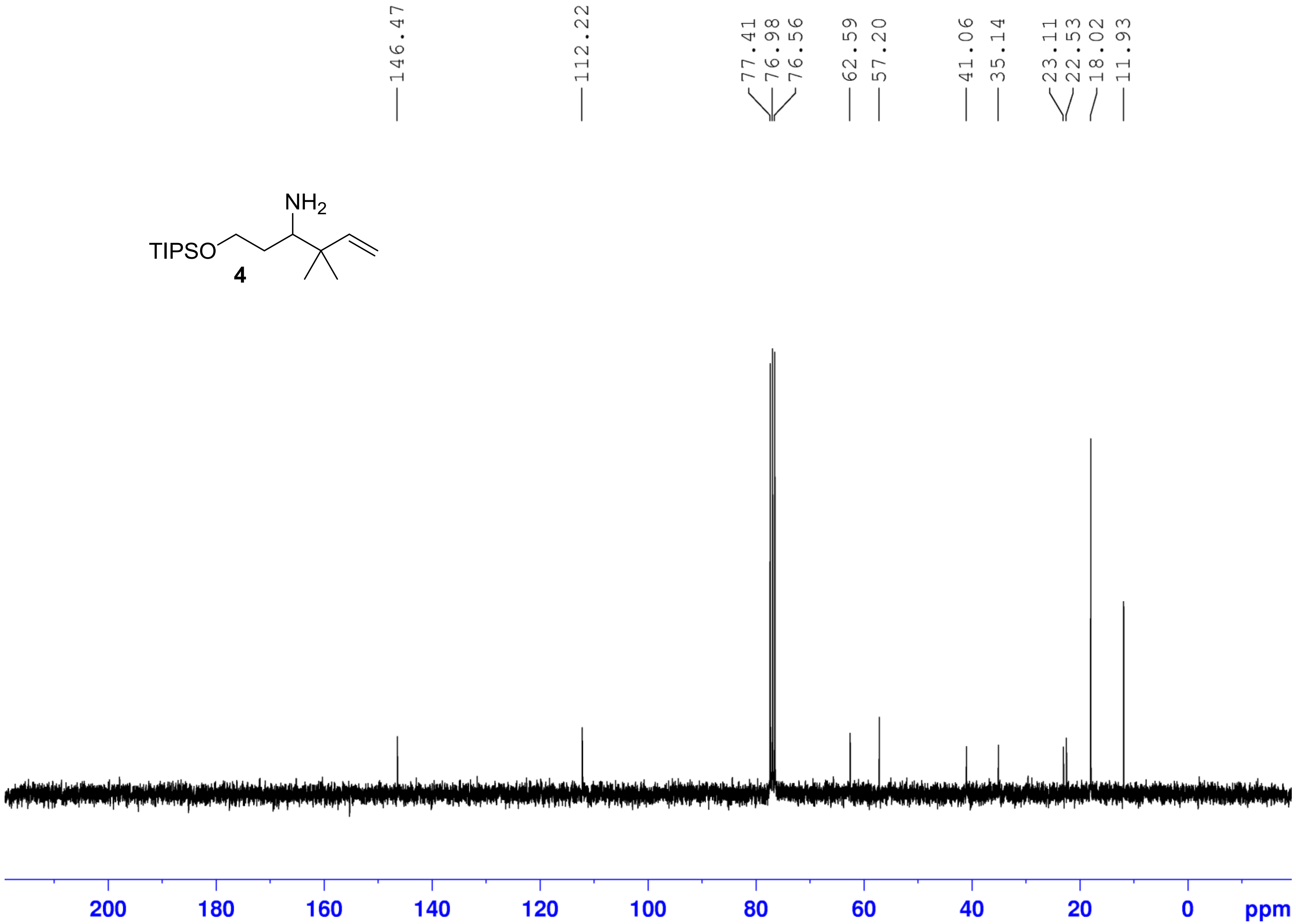

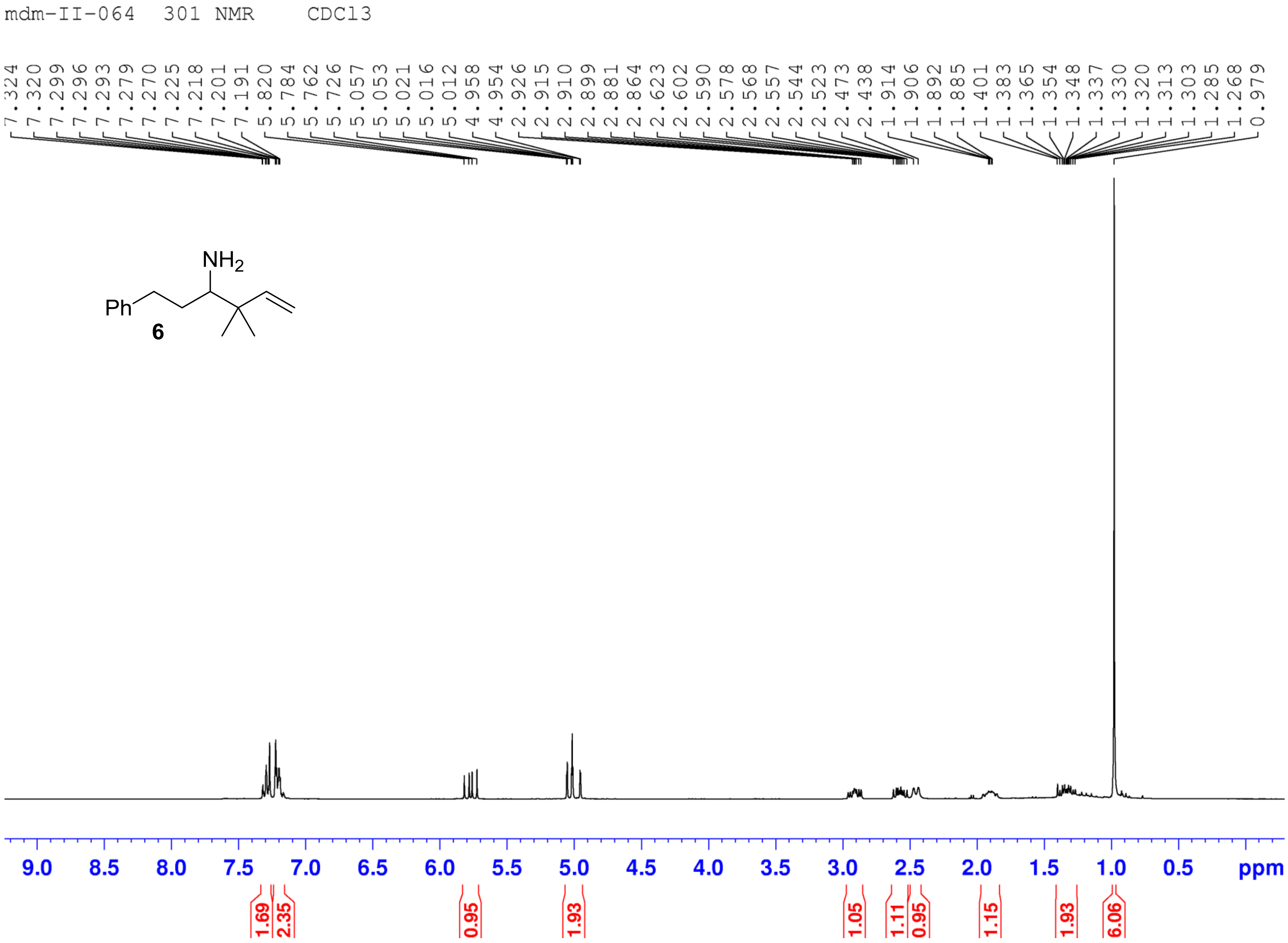
mdm-II-064 301 NMR CDCl3
7.324 7.320 7.299 7.296 7.293 7.279 7.270 7.225 7.218 7.201 7.191 5.820 5.784 5.762 5.726 5.057 5.053 5.021 5.016 5.012 4.958 4.954 2.926 2.915 2.910 2.899 2.881 2.864 2.623 2.602 2.590 2.578 2.568 2.557 2.544 2.523 2.473 2.438 1.914 1.906 1.892 1.885 1.401 1.383 1.365 1.354 1.348 1.337 1.330 1.320 1.313 1.303 1.285 1.268 0.979
NH2
Ph
6
9.0 8.5 8.0 7.5 7.0 6.5 6.0 5.5 5.0 4.5 4.0 3.5 3.0 2.5 2.0 1.5 1.0 0.5 ppm
1.69 2.35 0.95 1.93 1.05 1.11 0.95 1.15 1.93 6.06

mdm-II-064 301 NMR CDCl3

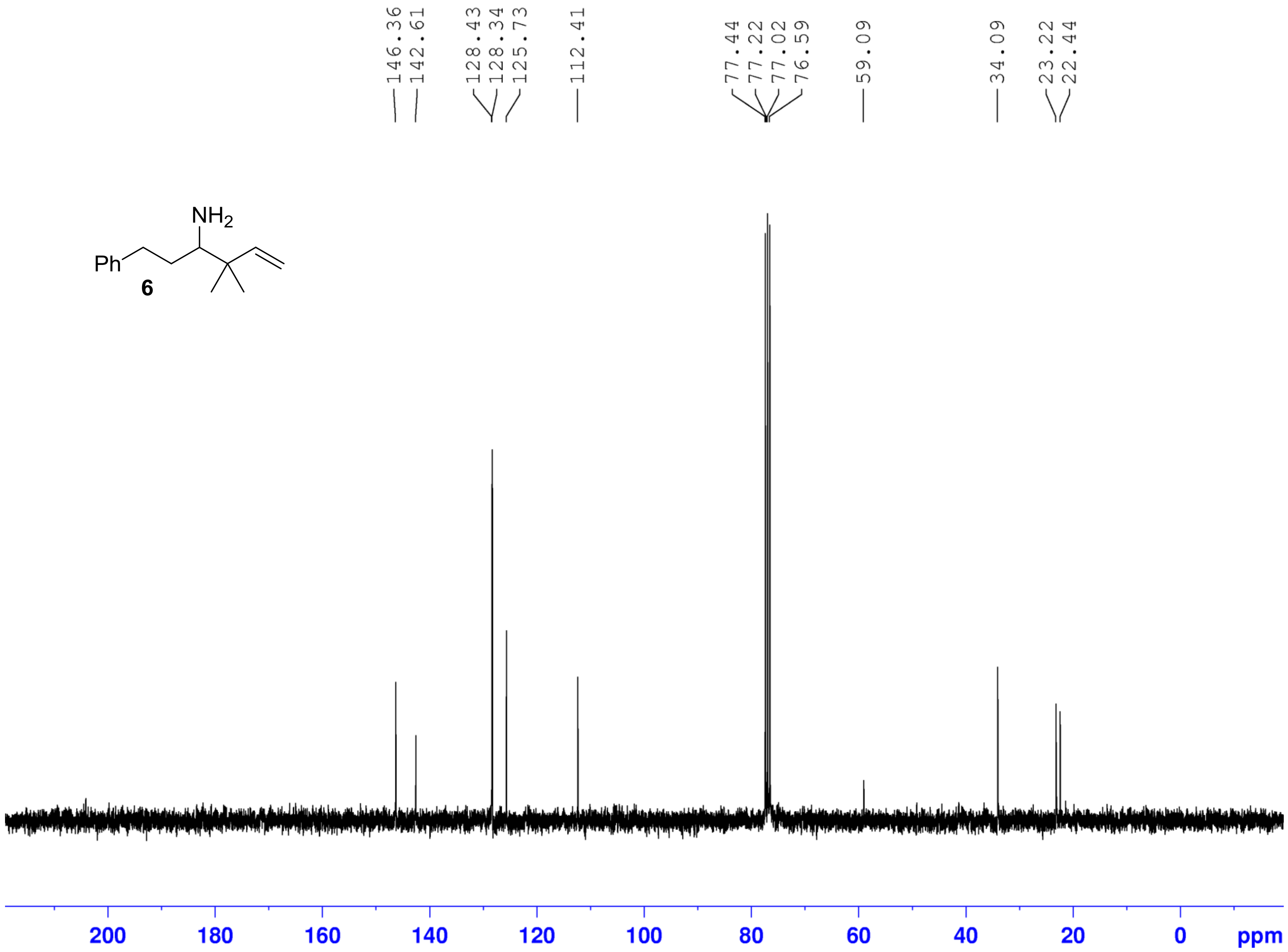

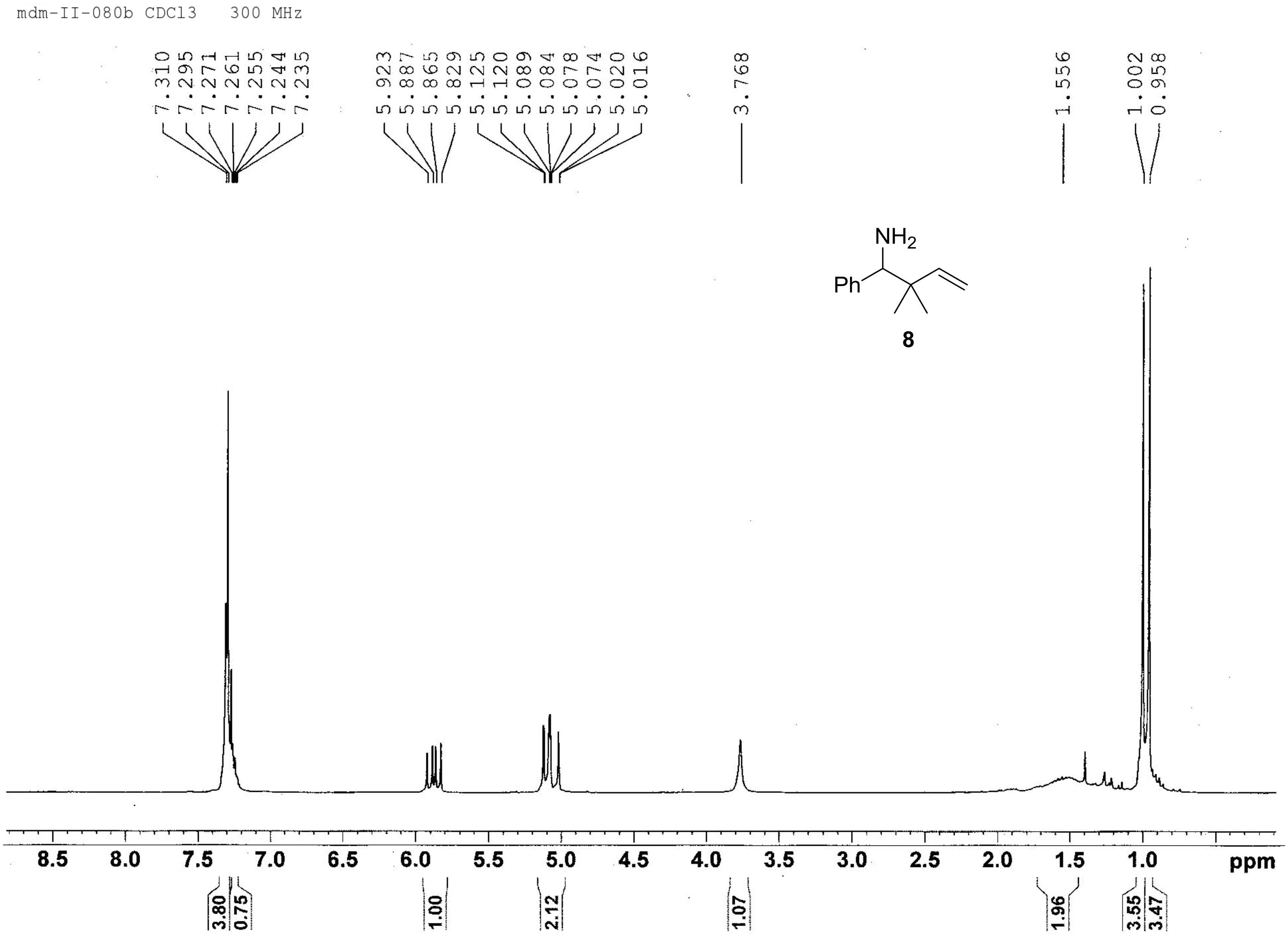
mdm-II-080b CDCl3 300 MHz
7.310 7.295 7.271 7.261 7.255 7.244 7.235
5.923 5.887 5.865 5.829 5.125 5.120 5.089 5.084 5.078 5.074 5.020 5.016
3.768
1.556
1.002 0.958
NH2
Ph
8
8.5 8.0 7.5 7.0 6.5 6.0 5.5 5.0 4.5 4.0 3.5 3.0 2.5 2.0 1.5 1.0 ppm
3.80 0.75 1.00 2.12 1.07 1.96 3.55 3.47

mdm-I-080b in CDCl3 301 NMR 8/31/07

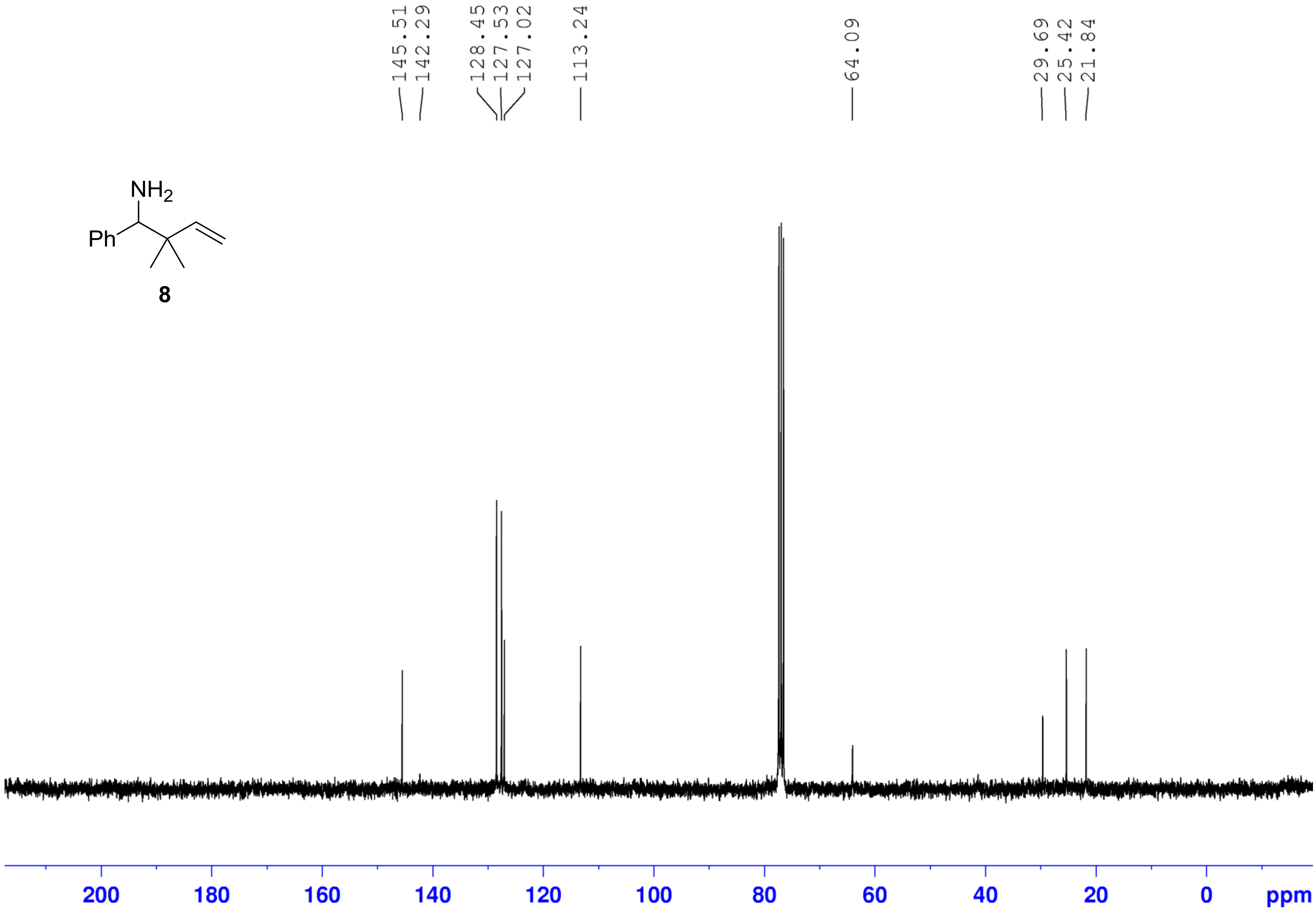

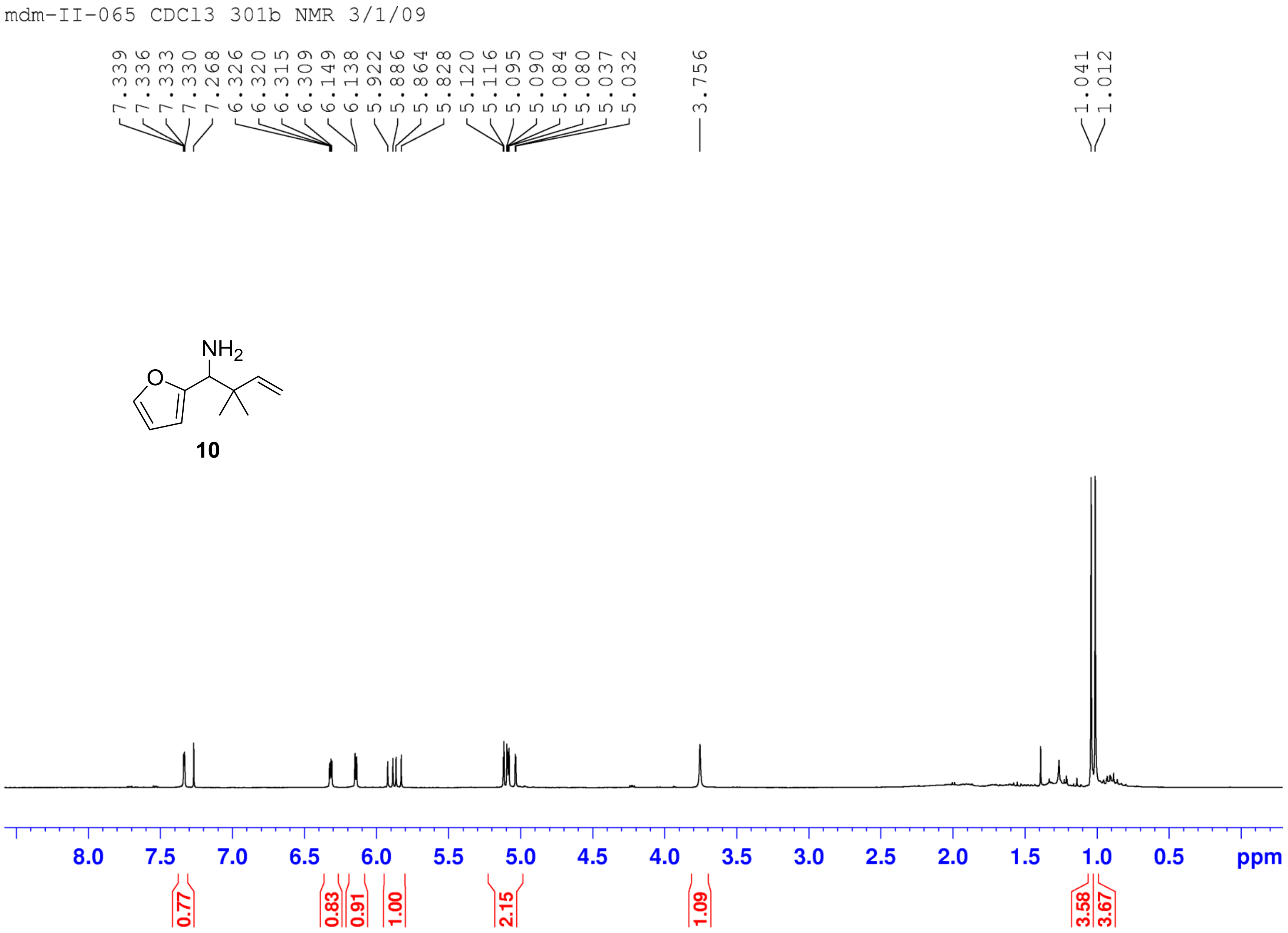
mdm-II-065 CDCl3 301b NMR 3/1/09
7.339
7.336
7.333
7.330
7.268
6.326
6.320
6.315
6.309
6.149
6.138
5.922
5.886
5.864
5.828
5.120
5.116
5.095
5.090
5.084
5.080
5.037
5.032
3.756
1.041
1.012
NH2
O
10
8.0
7.5
7.0
6.5
6.0
5.5
5.0
4.5
4.0
3.5
3.0
2.5
2.0
1.5
1.0
0.5
ppm
0.77
0.83
0.91
1.00
2.15
1.09
3.58
3.67

mdm-II-065 CDCl3 301b NMR 3/1/09

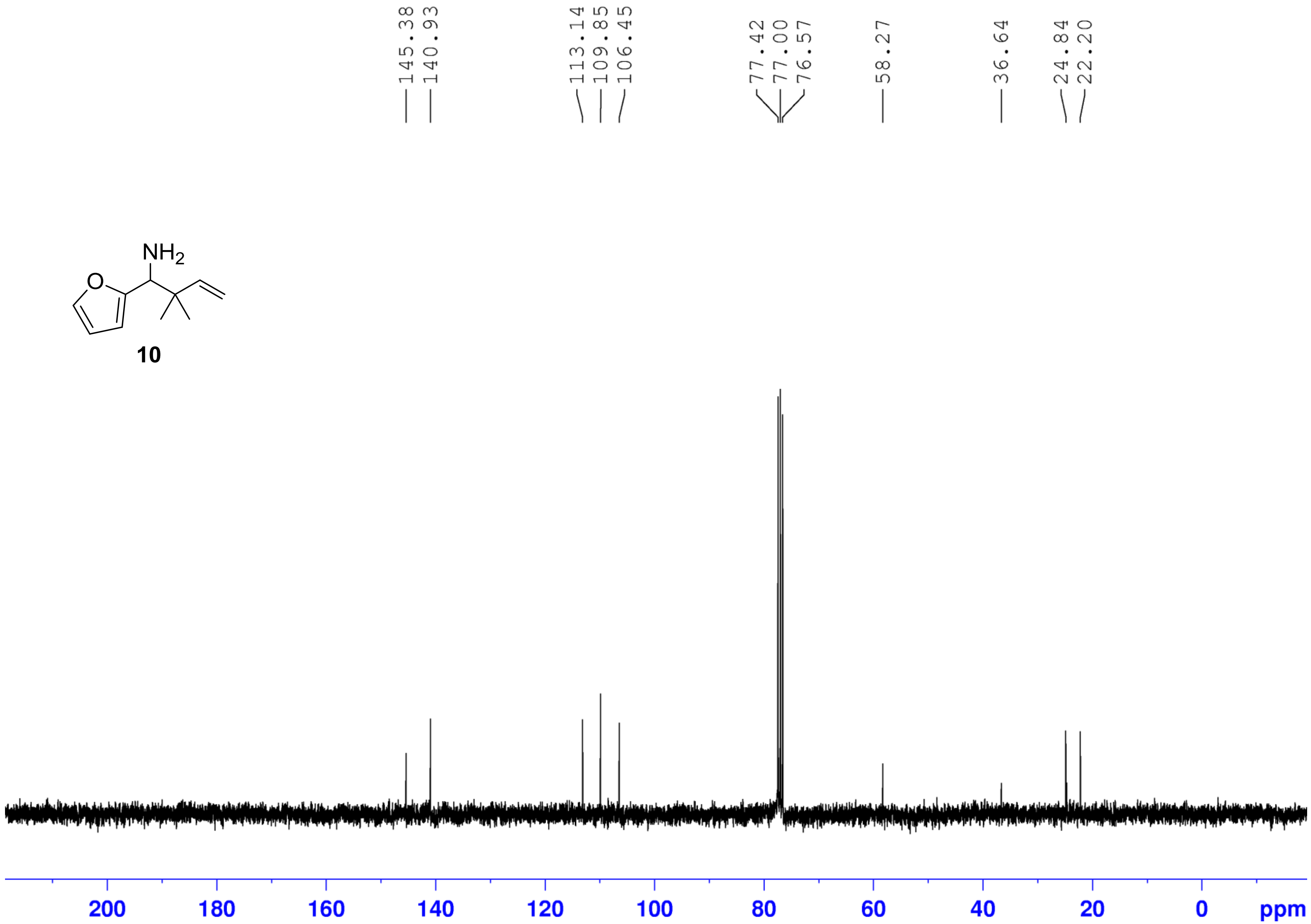

mdm-Ie-084 CDCl3 301NMR

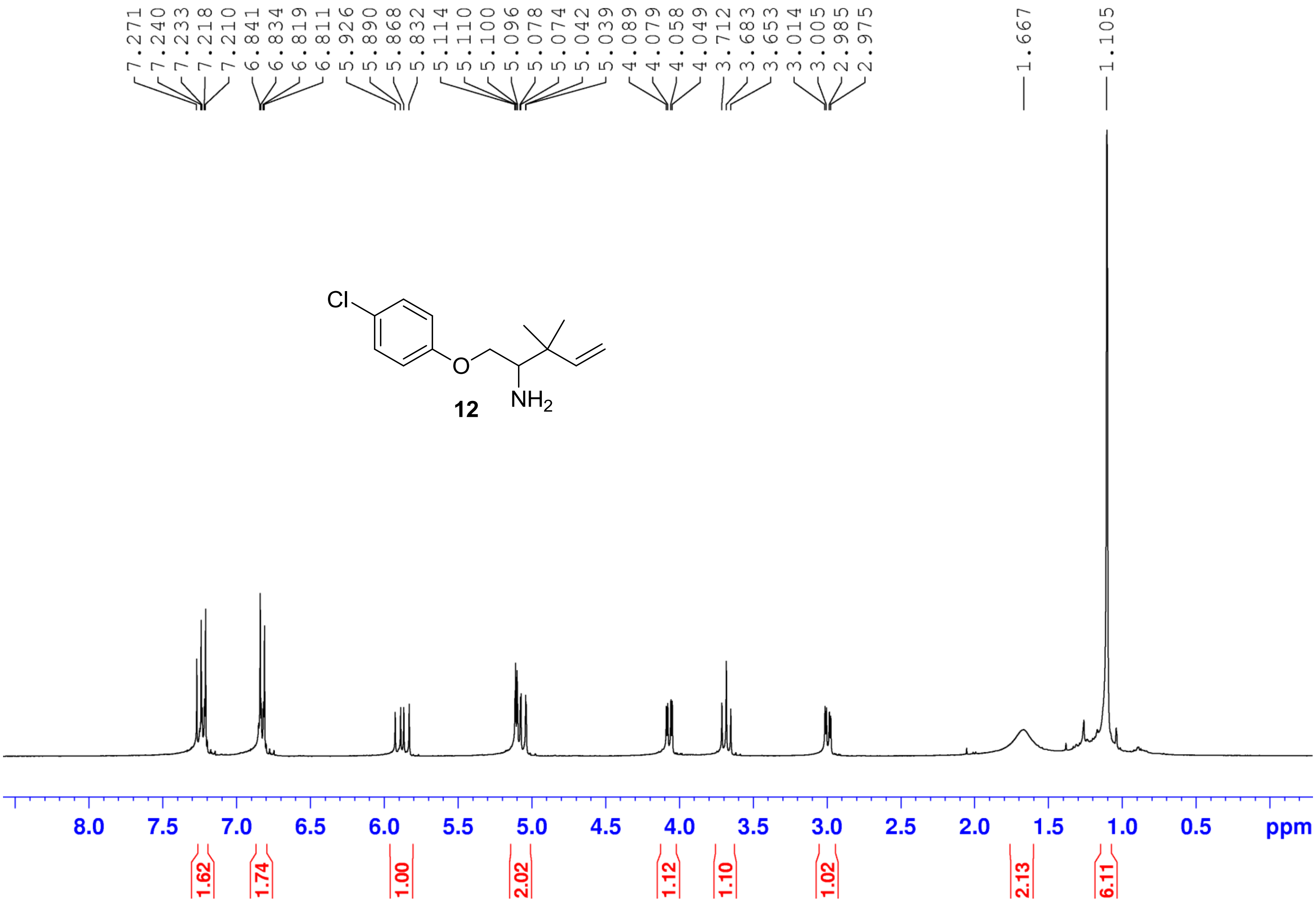

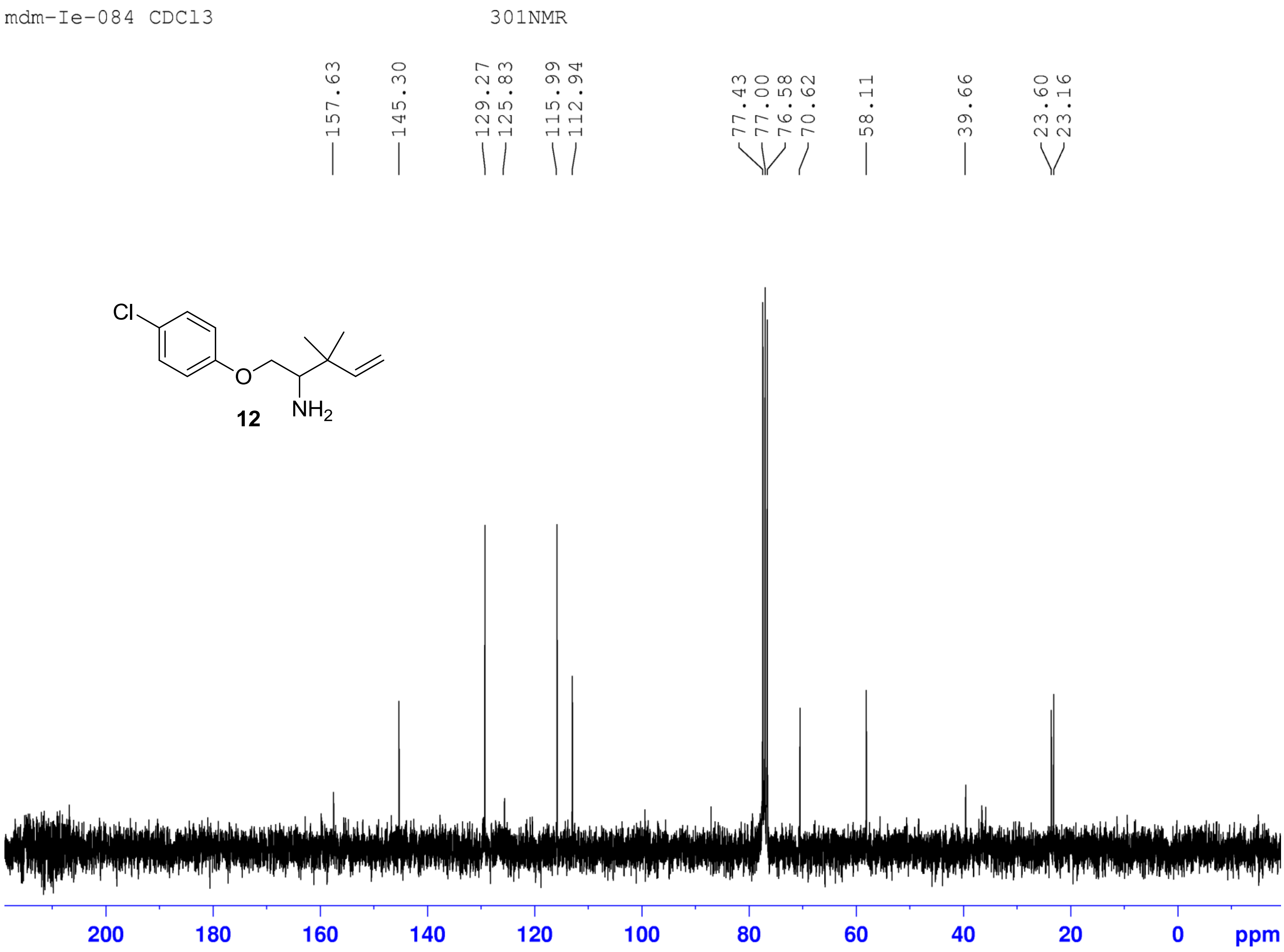
mdm-Ie-084 CDCl3
301NMR
157.63
145.30
129.27
125.83
115.99
112.94
77.43
77.00
76.58
70.62
58.11
39.66
23.60
23.16
Cl
O
NH2
12
200
180
160
140
120
100
80
60
40
20
0
ppm

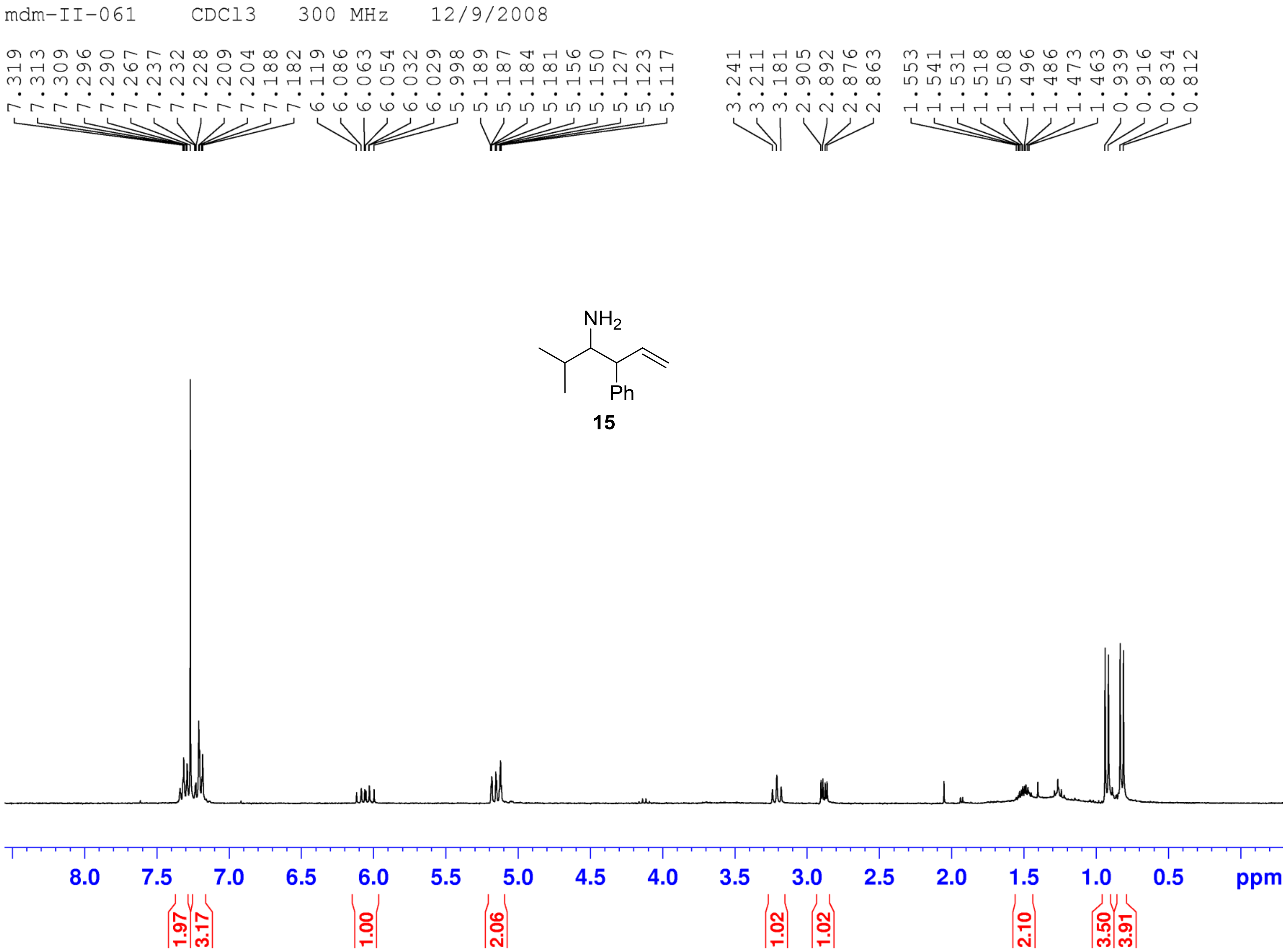
mdm-II-061 CDCl3 300 MHz 12/9/2008
7.319 7.313 7.309 7.296 7.290 7.267 7.237 7.232 7.228 7.209 7.204 7.188 7.182 6.119 6.086 6.063 6.054 6.032 6.029 5.998 5.189 5.187 5.184 5.181 5.156 5.150 5.127 5.123 5.117
3.241 3.211 3.181 2.905 2.892 2.876 2.863
1.553 1.541 1.531 1.518 1.508 1.496 1.486 1.473 1.463 0.939 0.916 0.834 0.812
NH2
Ph
15
8.0 7.5 7.0 6.5 6.0 5.5 5.0 4.5 4.0 3.5 3.0 2.5 2.0 1.5 1.0 0.5 ppm
1.97 3.17 1.00 2.06 1.02 1.02 2.10 3.50 3.91

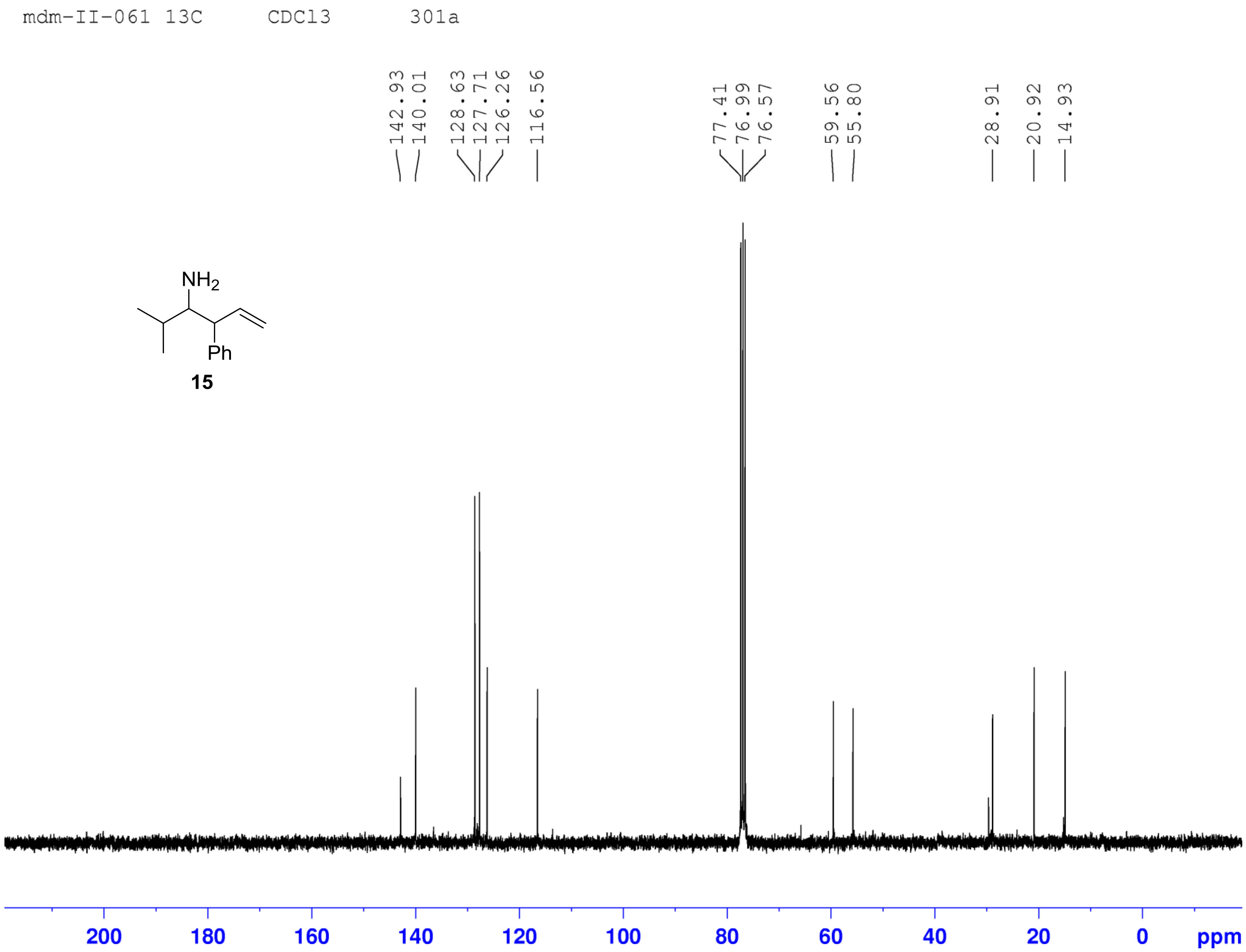
mdm-II-061 13C CDCl3 301a
142.93
140.01
128.63
127.71
126.26
116.56
77.41
76.99
76.57
59.56
55.80
28.91
20.92
14.93
NH2
Ph
15
200
180
160
140
120
100
80
60
40
20
0
ppm

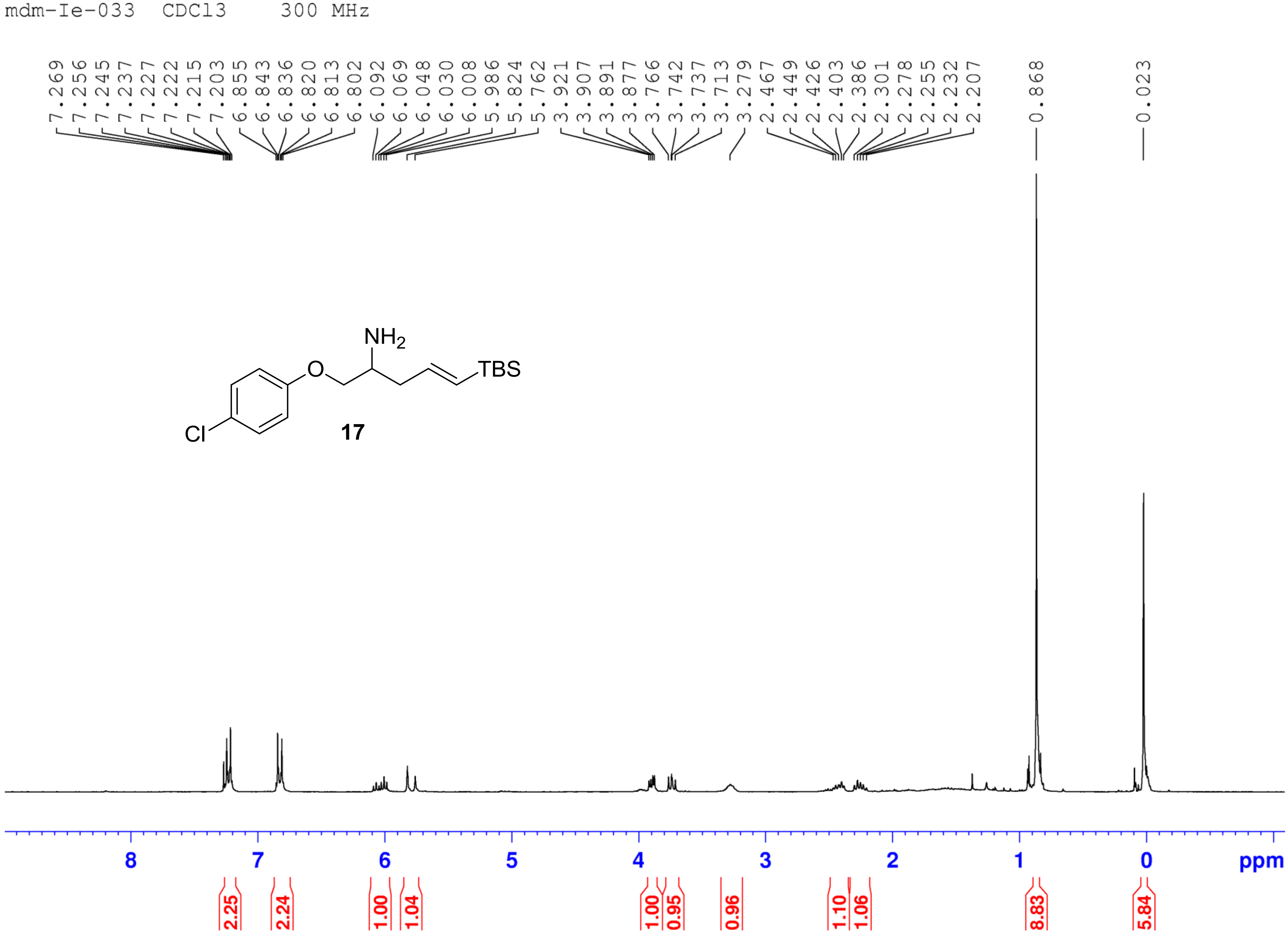
mdm-Ie-033 CDCl3 300 MHz
7.269
7.256
7.245
7.237
7.227
7.222
7.215
7.203
6.855
6.843
6.836
6.820
6.813
6.802
6.092
6.069
6.048
6.030
6.008
5.986
5.824
5.762
3.921
3.907
3.891
3.877
3.766
3.742
3.737
3.713
3.279
2.467
2.449
2.426
2.403
2.386
2.301
2.278
2.255
2.232
2.207
0.868
0.023
NH2
O
TBS
Cl
17
8
7
6
5
4
3
2
1
0
ppm
2.25
2.24
1.00
1.04
1.00
0.95
0.96
1.10
1.06
8.83
5.84

mdm-Ie-033 13C NMR 301

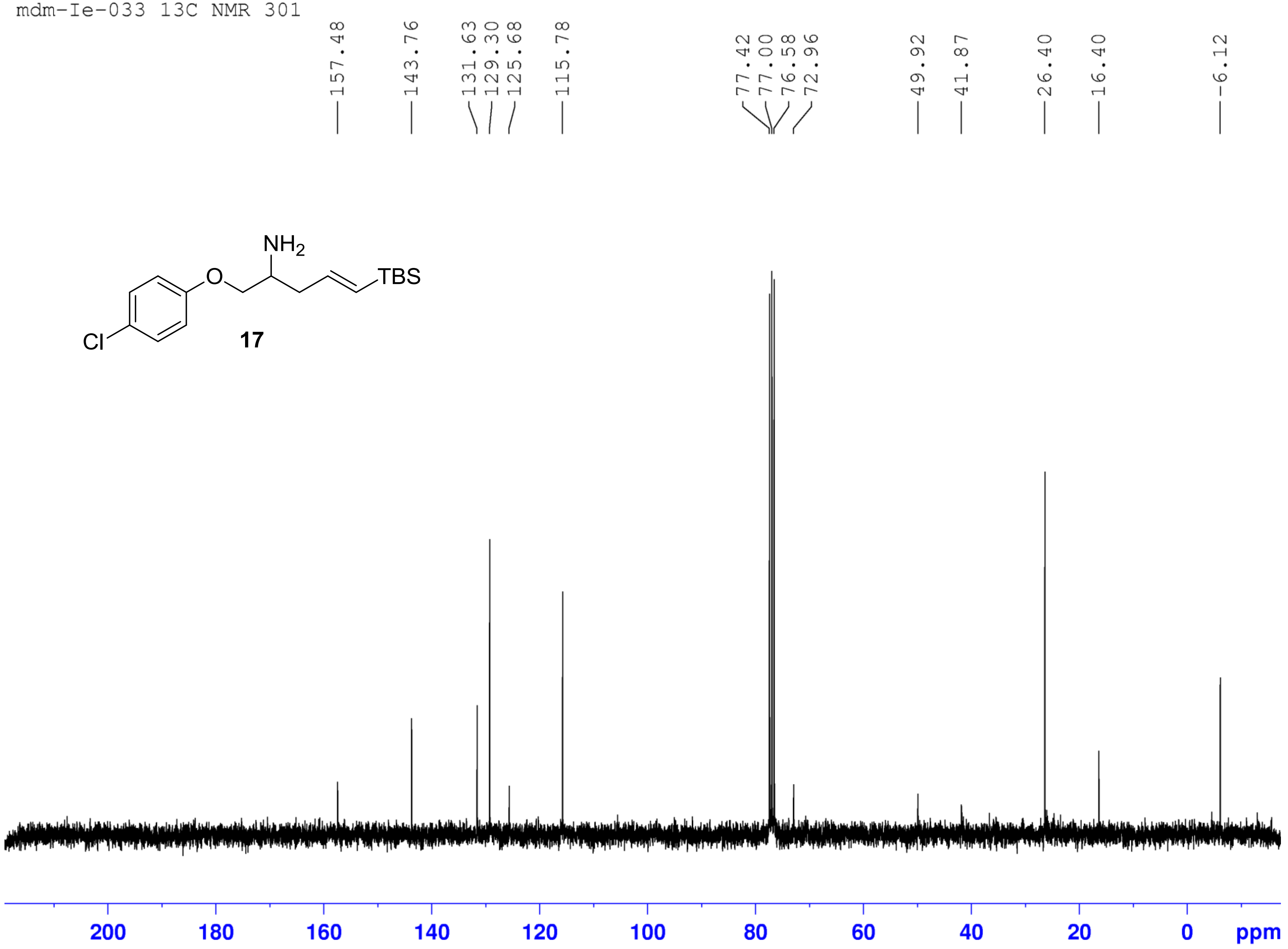


**17**

mdm-II-039 in CDCl3 301 NMR

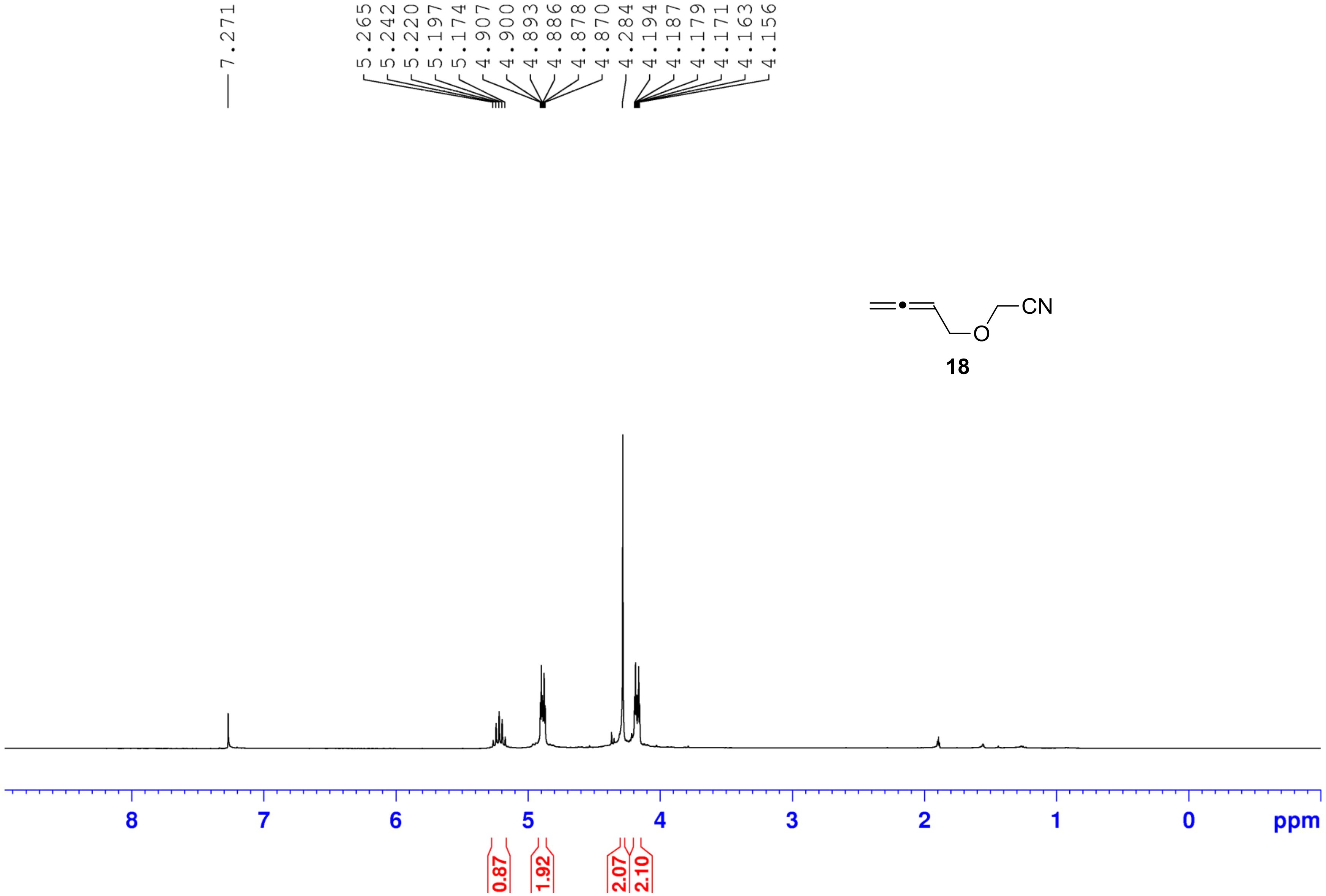

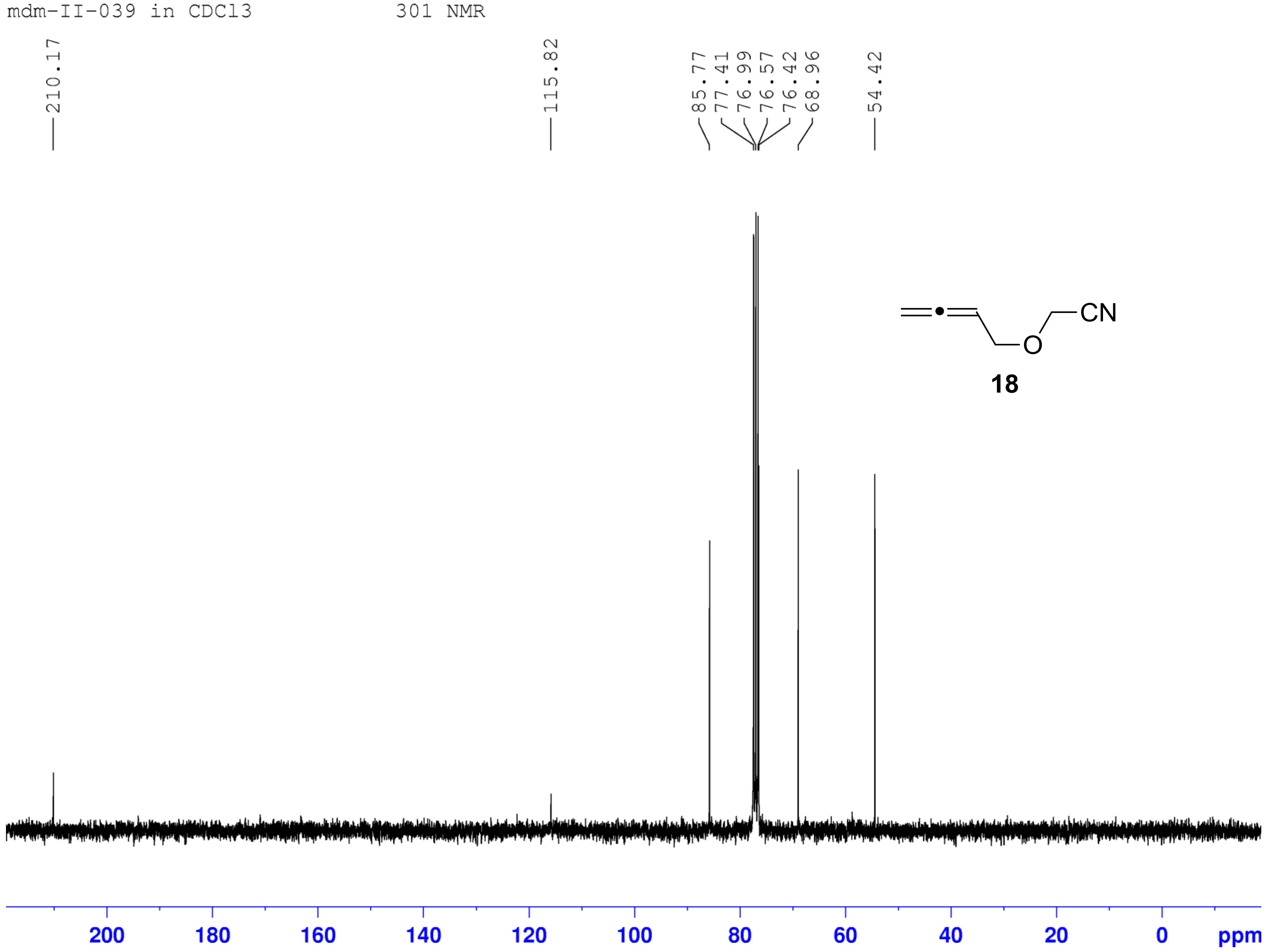
mdm-II-039 in CDCl3
301 NMR
210.17
115.82
85.77
77.41
76.99
76.57
76.42
68.96
54.42
CN
O
18
200
180
160
140
120
100
80
60
40
20
0
ppm

mdm-II-020 1H CDCl3

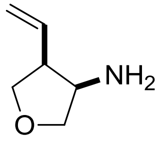

**19**

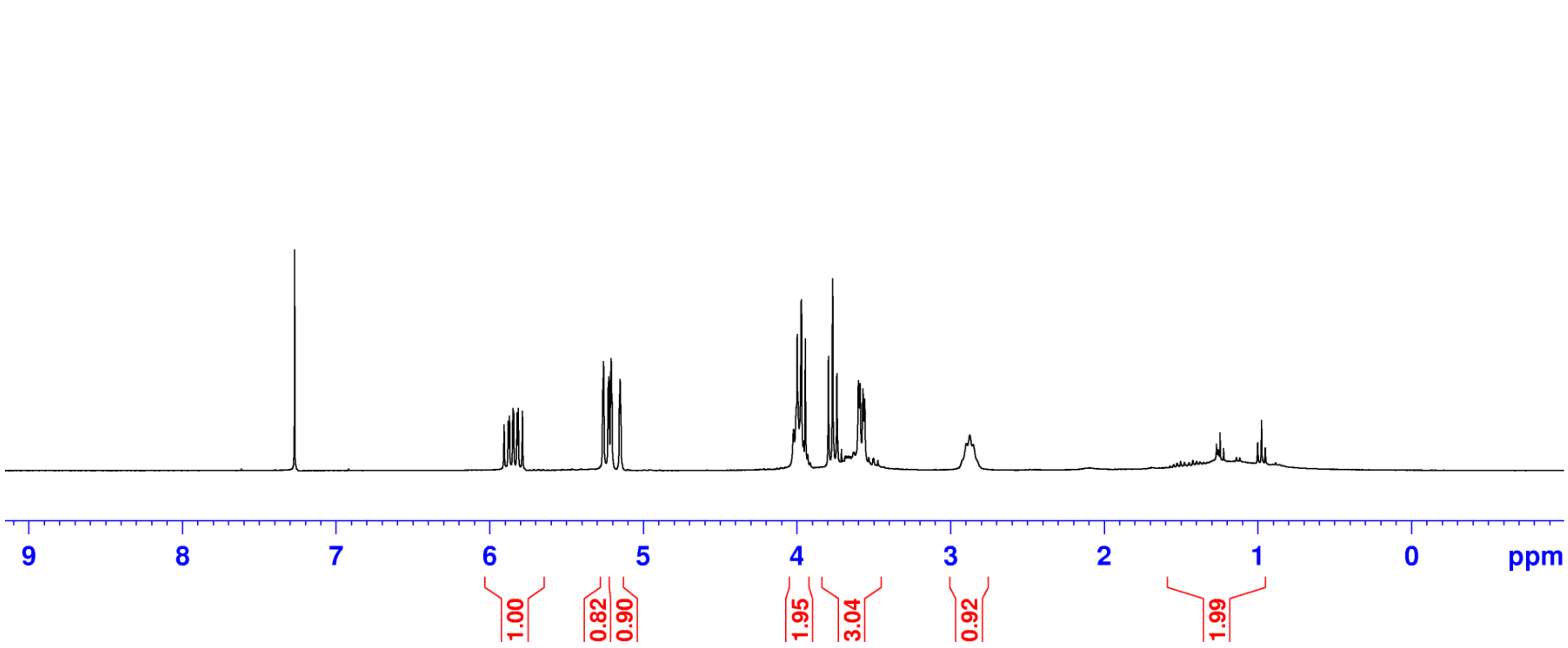

C-5 amine

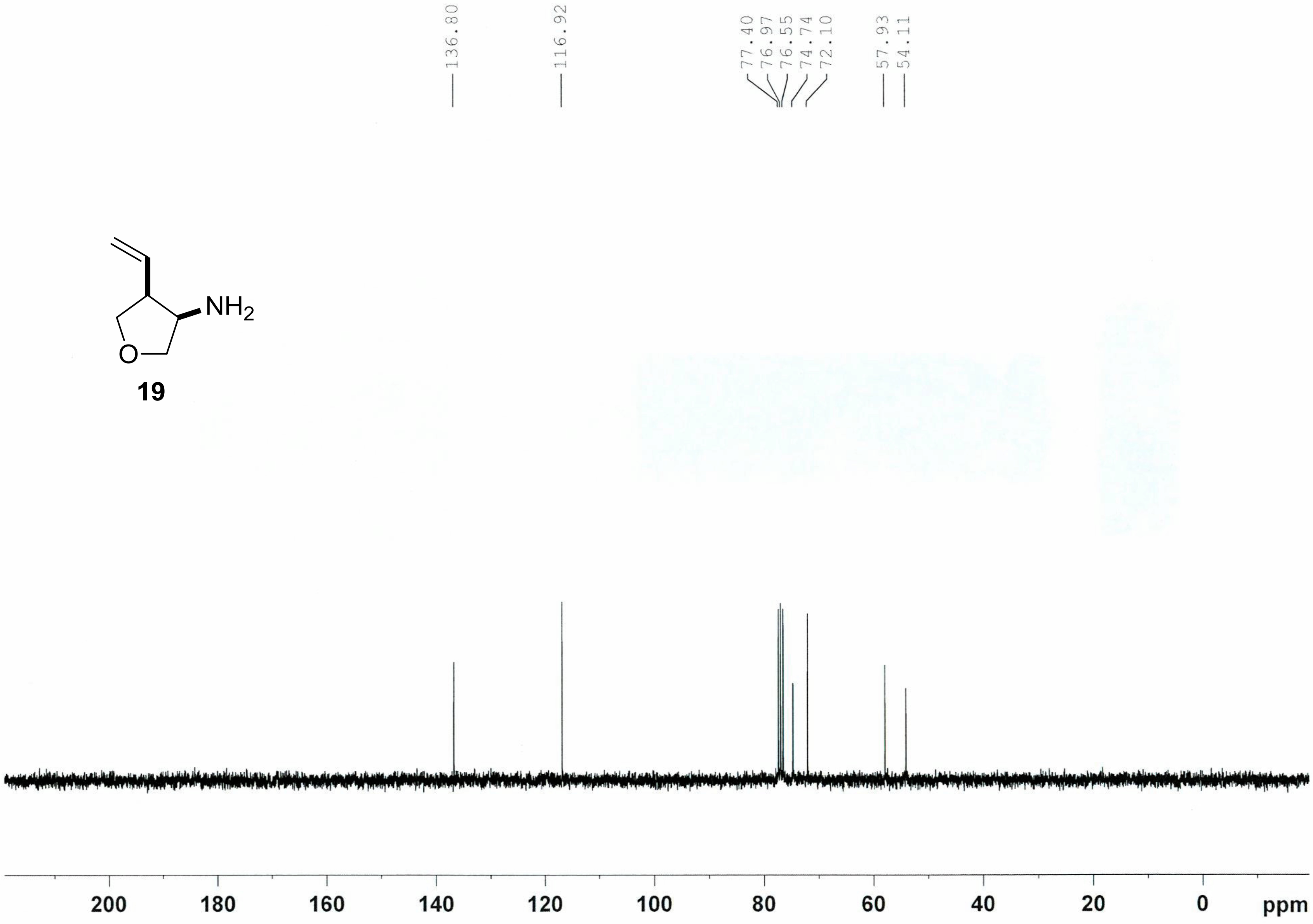
136.80
116.92
77.40
76.97
76.55
74.74
72.10
57.93
54.11
NH2
O
19
200
180
160
140
120
100
80
60
40
20
0
ppm

mdm-II-039 1H CDCl3

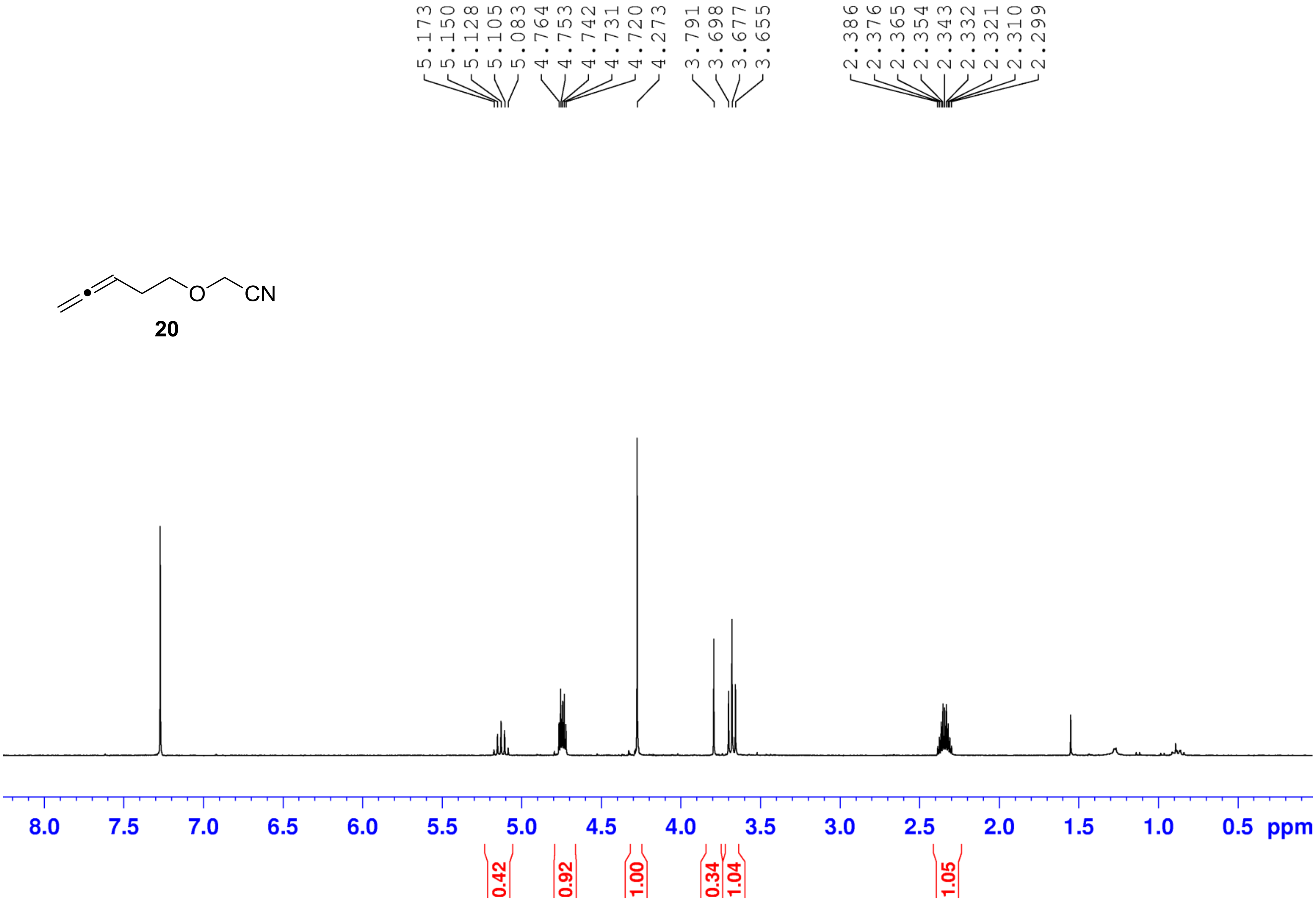

mdm-II-039  13C CDCl3

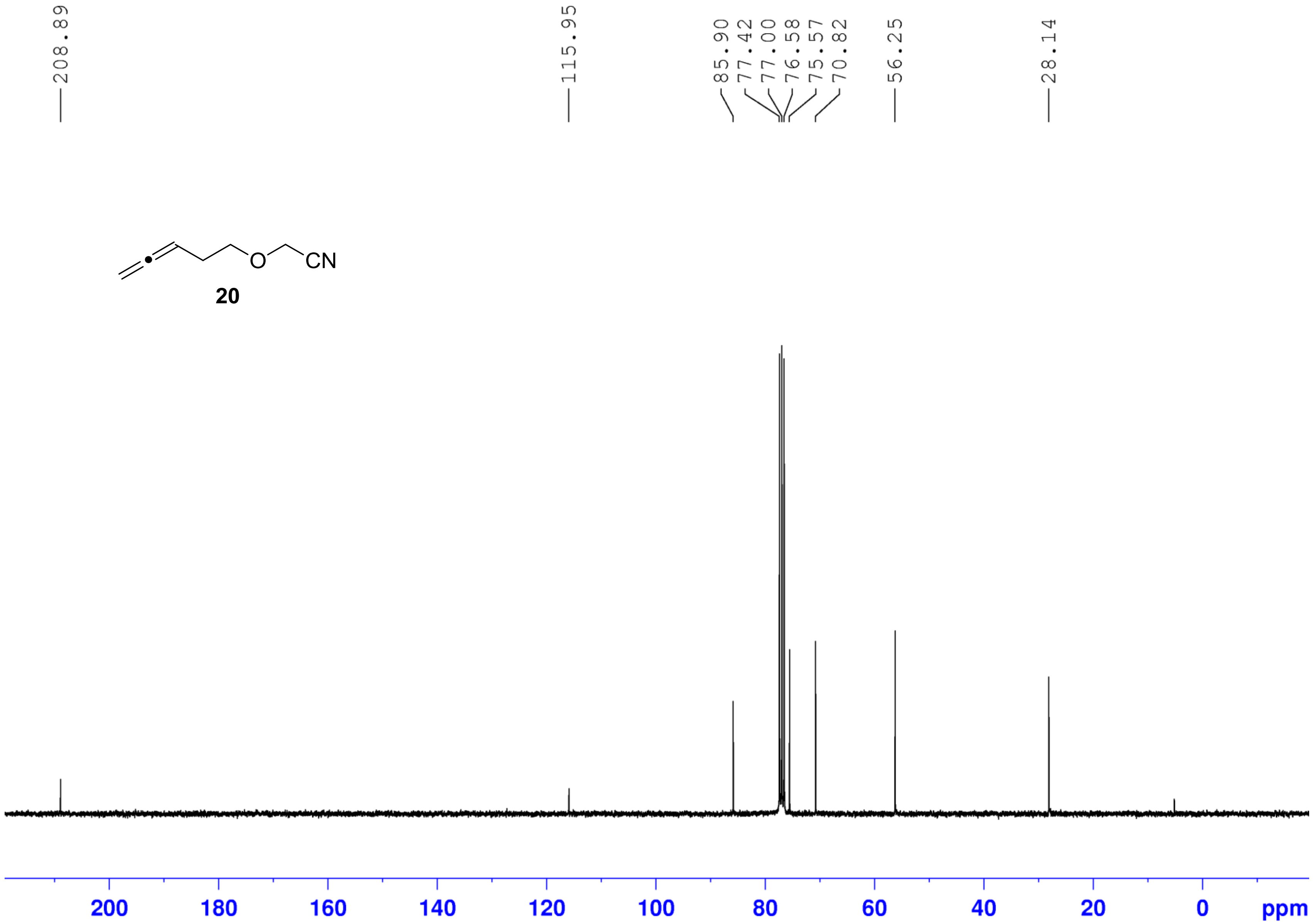

mdm-II-043 1H CDCl3 301b

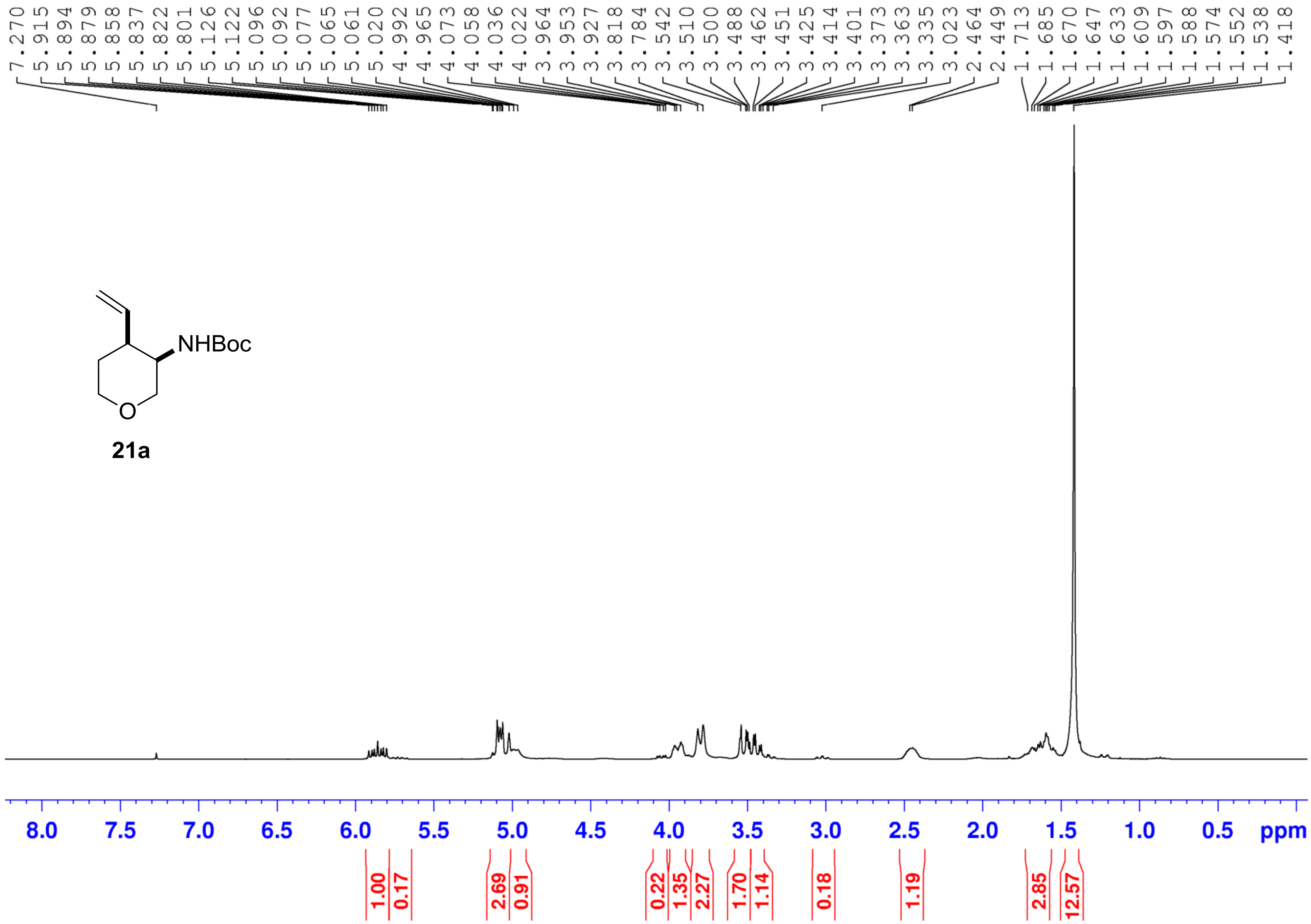

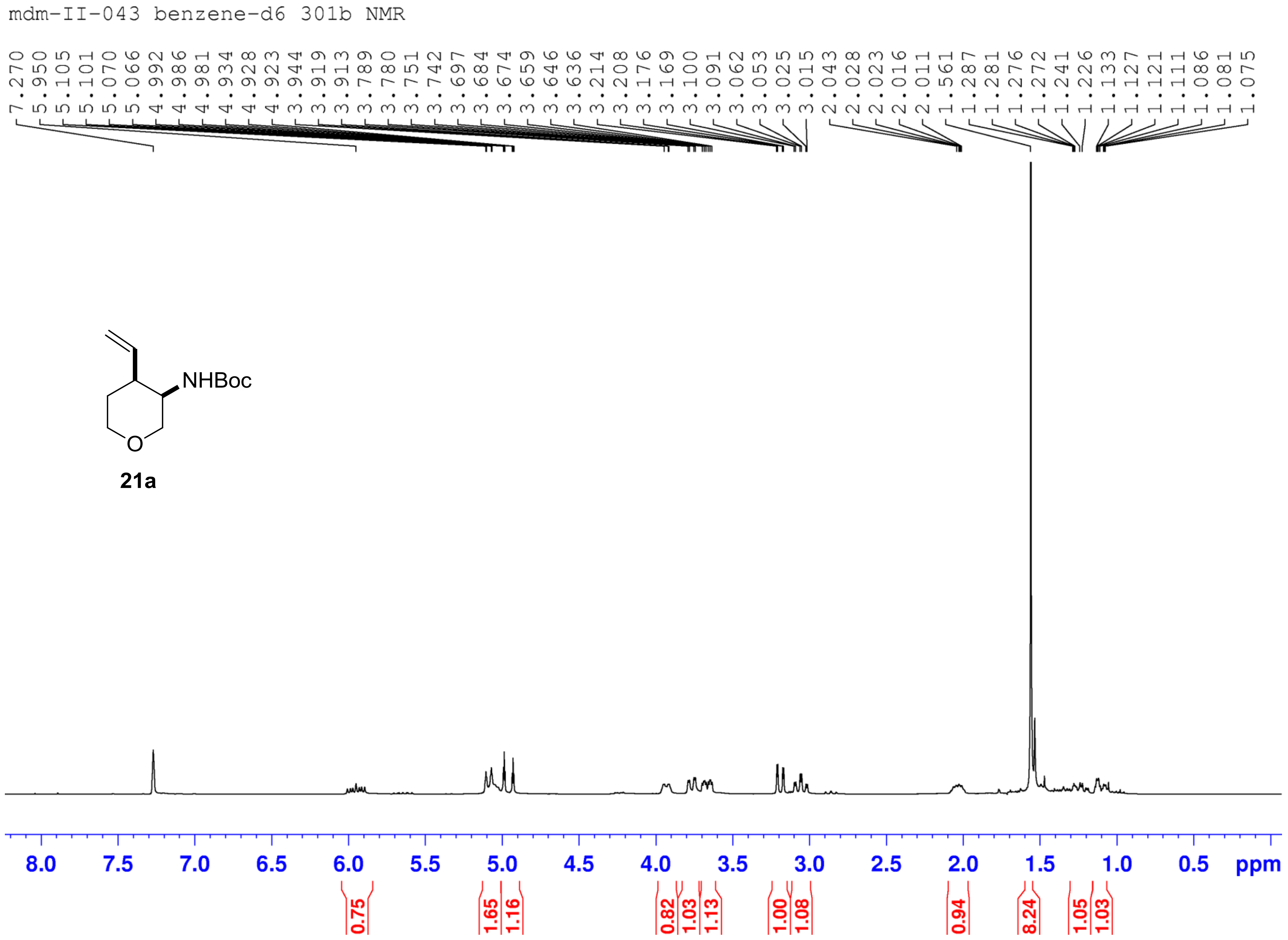
mdm-II-043 benzene-d6 301b NMR
7.270
5.950
5.105
5.101
5.070
5.066
4.992
4.986
4.981
4.934
4.928
4.923
3.944
3.919
3.913
3.789
3.780
3.751
3.742
3.697
3.684
3.674
3.659
3.646
3.636
3.214
3.208
3.176
3.169
3.100
3.091
3.062
3.053
3.025
3.015
2.043
2.028
2.023
2.016
2.011
1.561
1.287
1.281
1.276
1.272
1.241
1.226
1.133
1.127
1.121
1.111
1.086
1.081
1.075
NHBoc
O
21a
8.0
7.5
7.0
6.5
6.0
5.5
5.0
4.5
4.0
3.5
3.0
2.5
2.0
1.5
1.0
0.5
ppm
0.75
1.65
1.16
0.82
1.03
1.13
1.00
1.08
0.94
8.24
1.05
1.03

mdm-II-043 13C CDCl3 301b crude

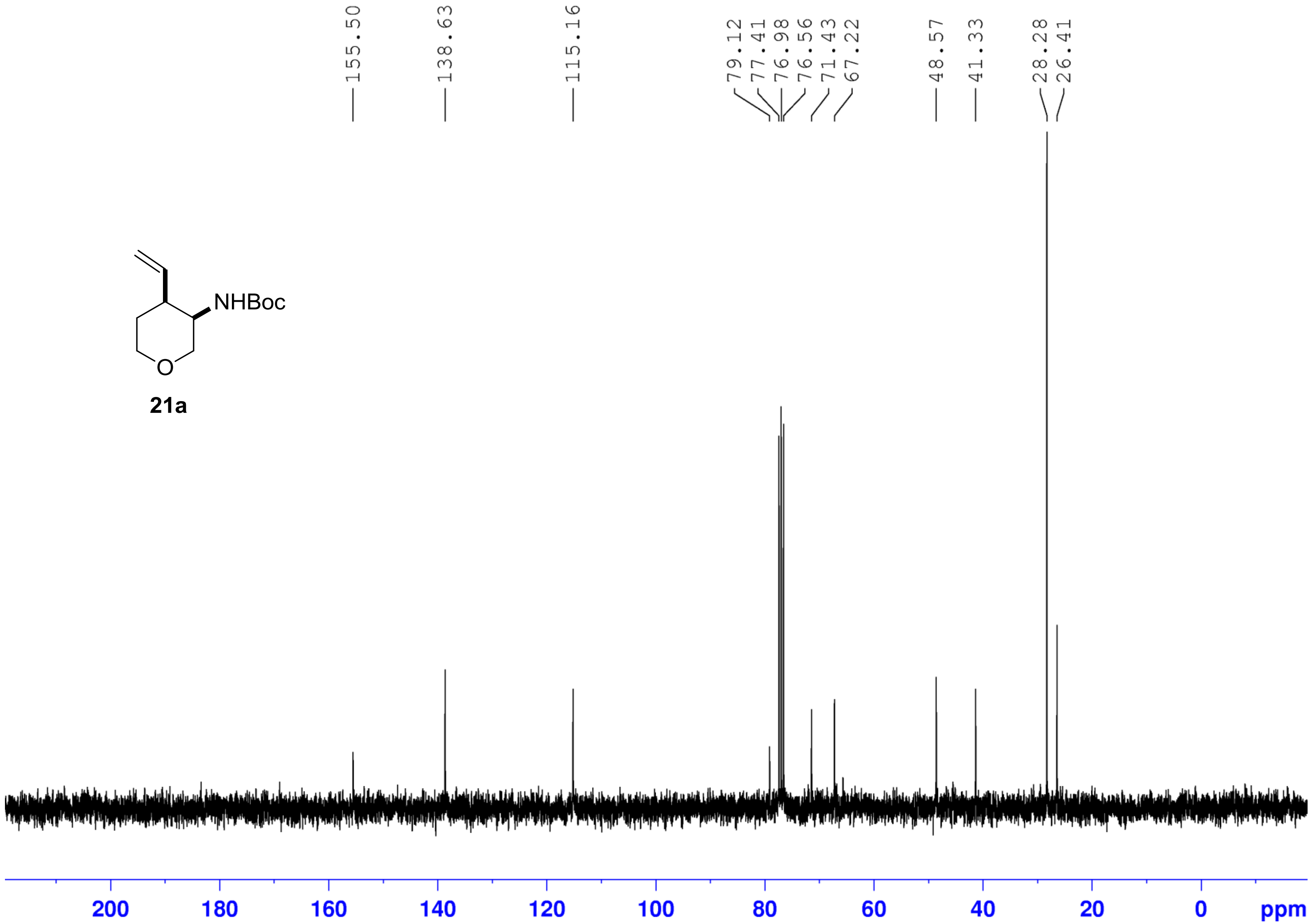


**21a**

mdm-II-079 CDCl3, 1H 300 MHz

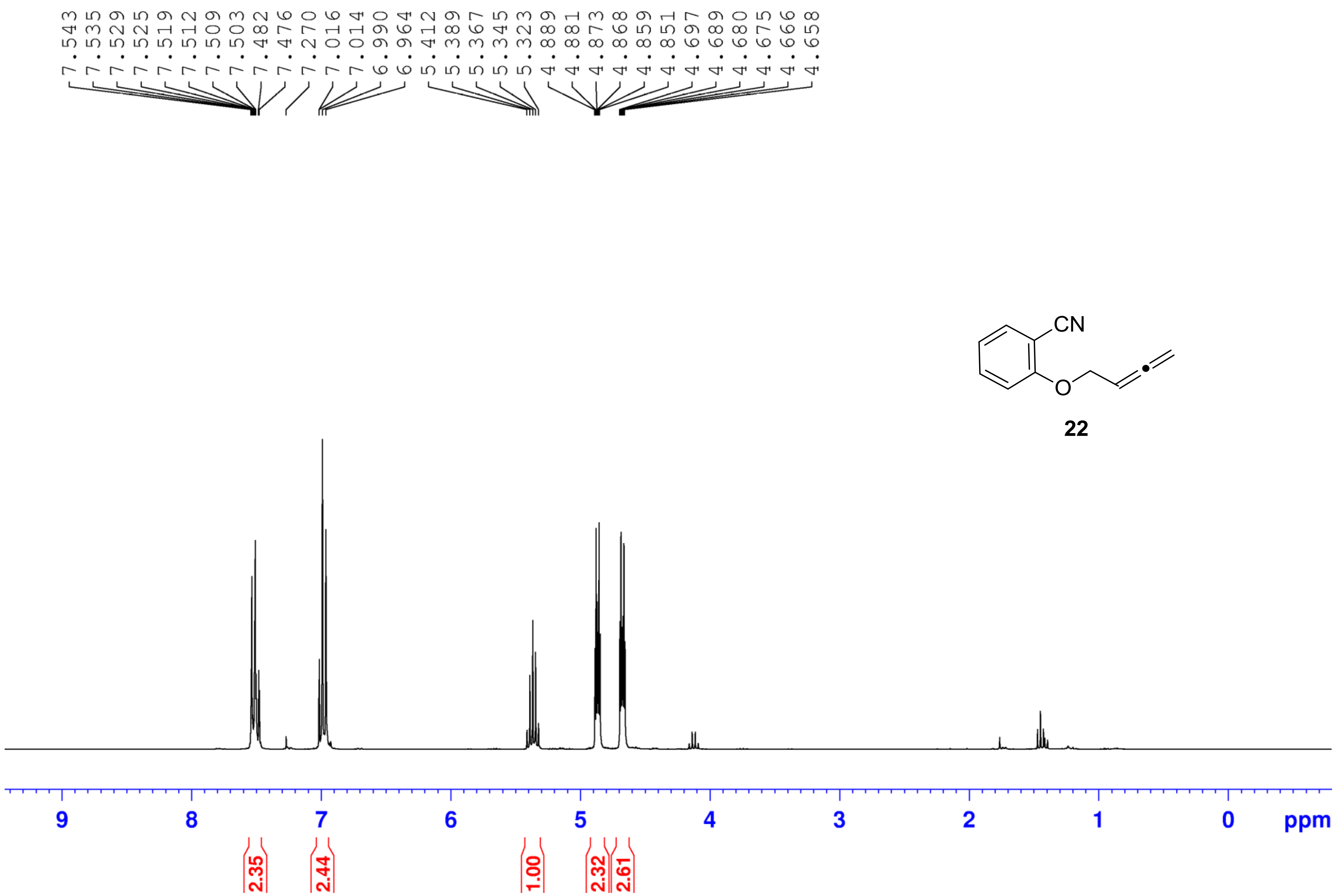


**22**

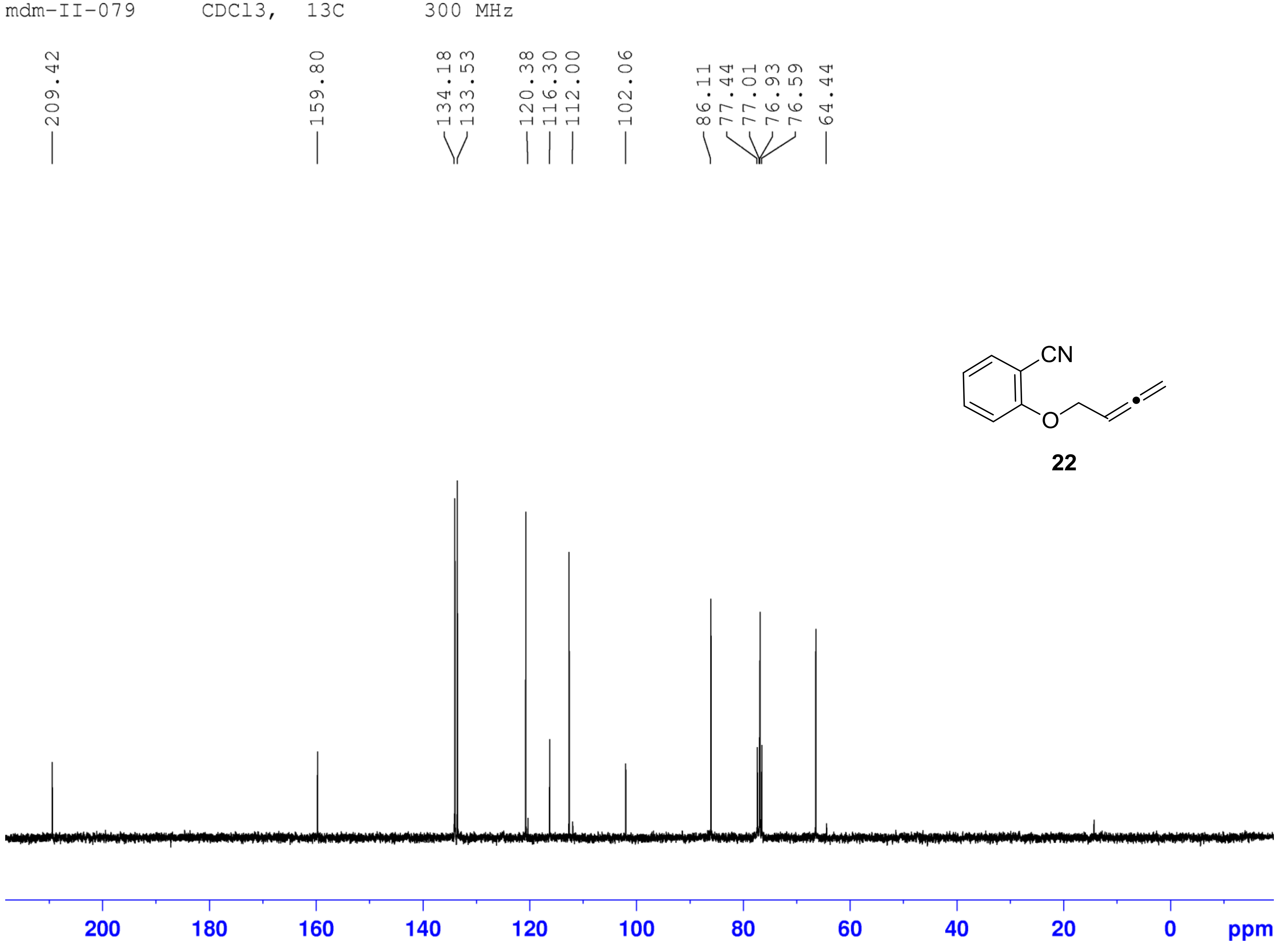
mdm-II-079 CDCl3, 13C 300 MHz
209.42
159.80
134.18
133.53
120.38
116.30
112.00
102.06
86.11
77.44
77.01
76.93
76.59
64.44
CN
O
22
200
180
160
140
120
100
80
60
40
20
0
ppm

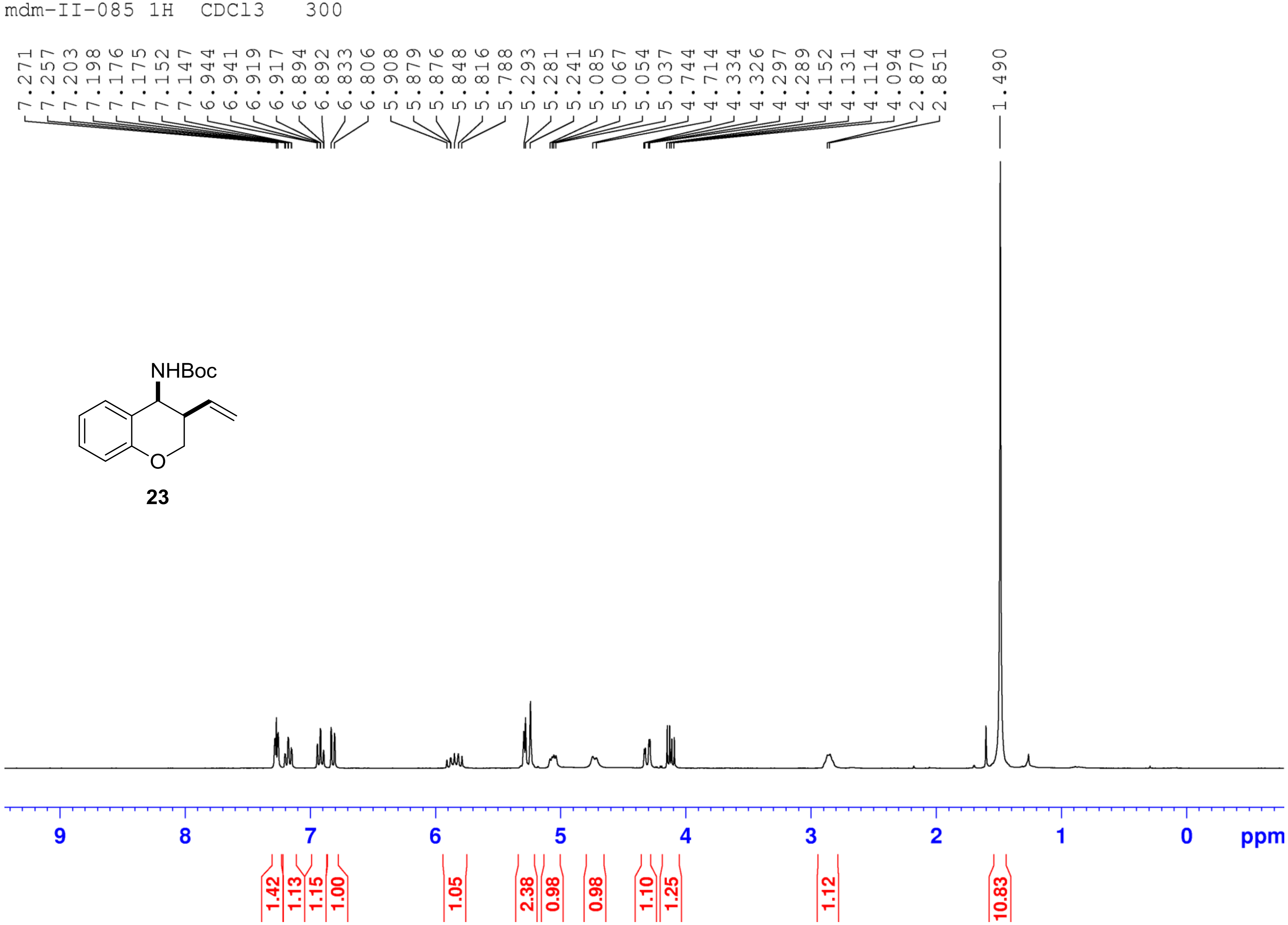
mdm-II-085 1H CDCl3 300
7.271 7.257 7.203 7.198 7.176 7.175 7.152 7.147 6.944 6.941 6.919 6.917 6.894 6.892 6.833 6.806 5.908 5.879 5.876 5.848 5.816 5.788 5.293 5.281 5.241 5.085 5.067 5.054 5.037 4.744 4.714 4.334 4.326 4.297 4.289 4.152 4.131 4.114 4.094 2.870 2.851 1.490
NHBoc
O
23
9 8 7 6 5 4 3 2 1 0 ppm
1.42 1.13 1.15 1.00 1.05 2.38 0.98 0.98 1.10 1.25 1.12 10.83

mdm-II-085 13C CDCl3 300

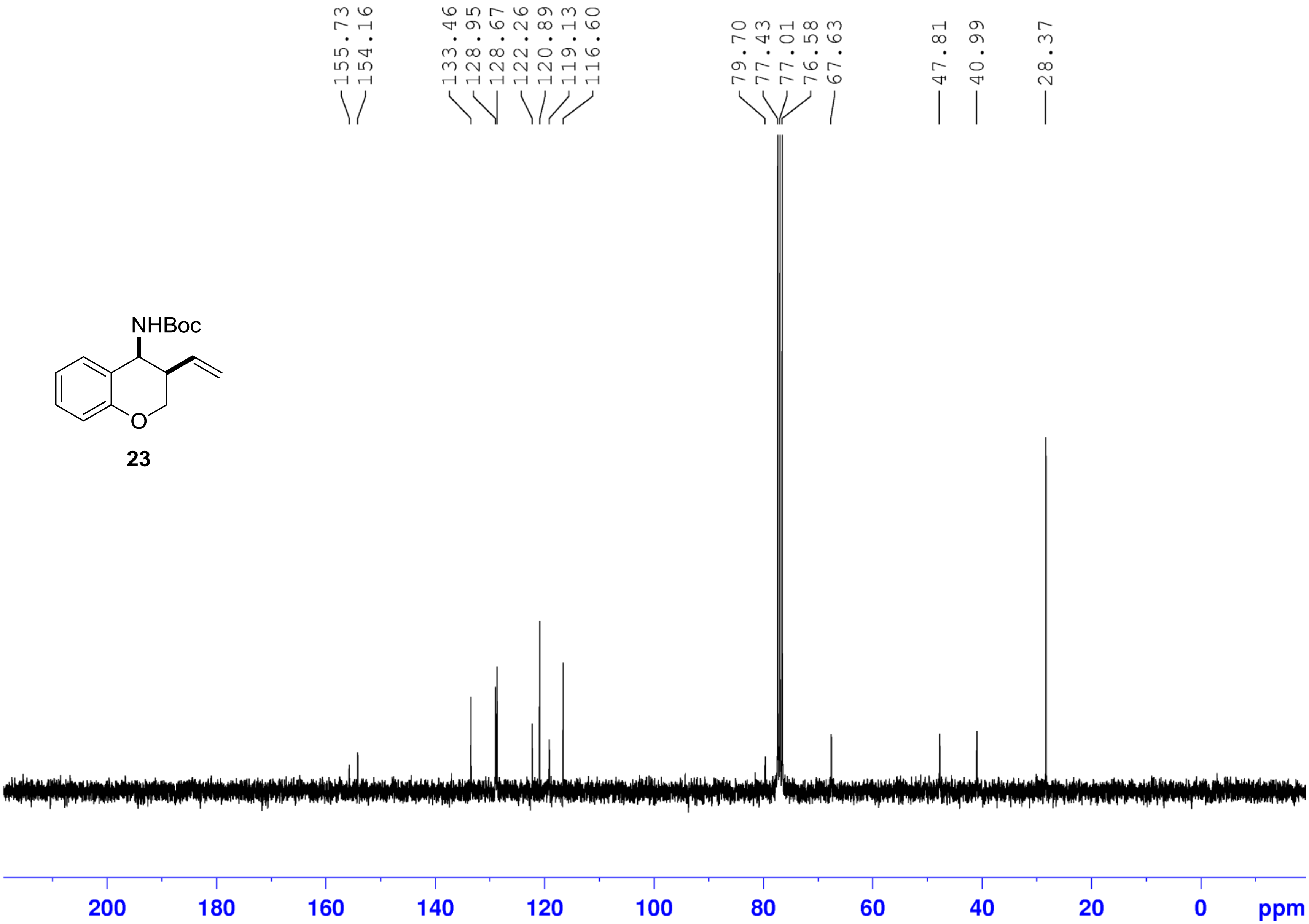


**23**

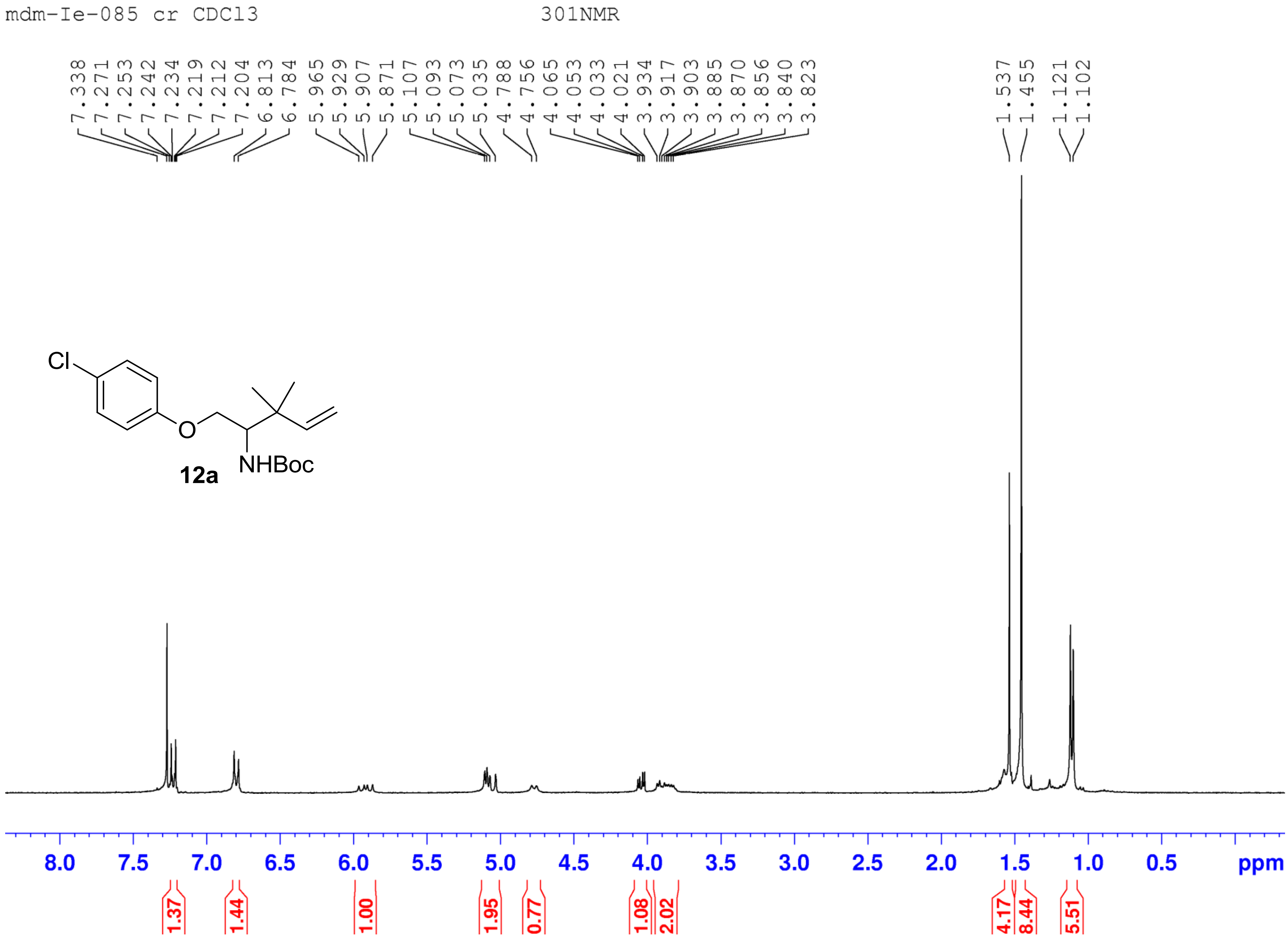
mdm-Ie-085 cr CDCl3
301NMR
7.338 7.271 7.253 7.242 7.234 7.219 7.212 7.204 6.813 6.784 5.965 5.929 5.907 5.871 5.107 5.093 5.073 5.035 4.788 4.756 4.065 4.053 4.033 4.021 3.934 3.917 3.903 3.885 3.870 3.856 3.840 3.823
1.537 1.455 1.121 1.102
Cl
O
NHBoc
12a
8.0 7.5 7.0 6.5 6.0 5.5 5.0 4.5 4.0 3.5 3.0 2.5 2.0 1.5 1.0 0.5 ppm
1.37 1.44 1.00 1.95 0.77 1.08 2.02 4.17 8.44 5.51

mdm-II-071 CDCl3 300 MHz

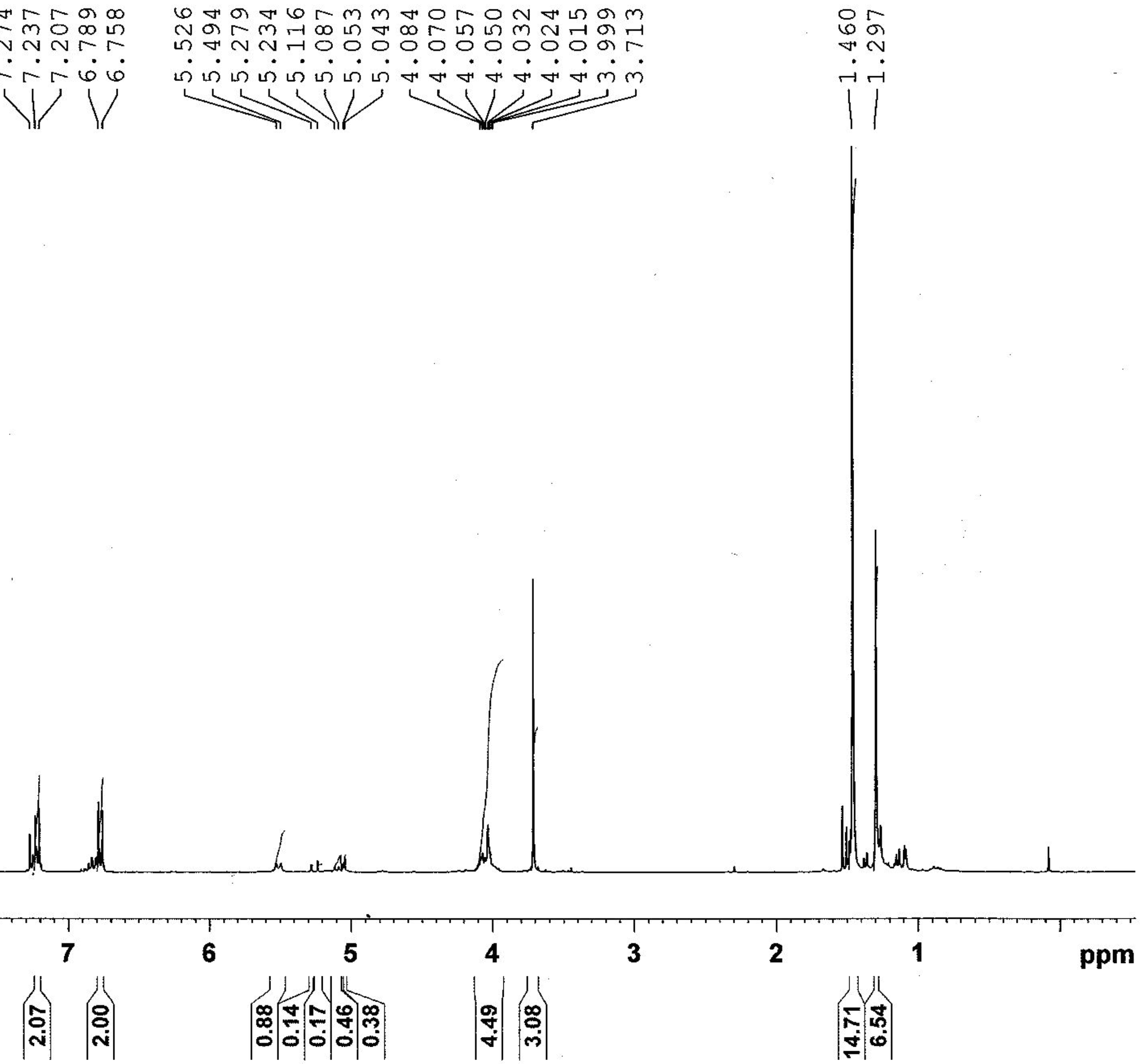


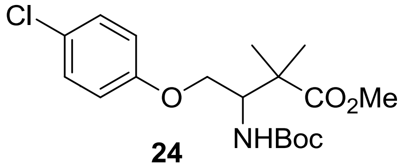


**24**

mdm-II-071 CDCl3 300

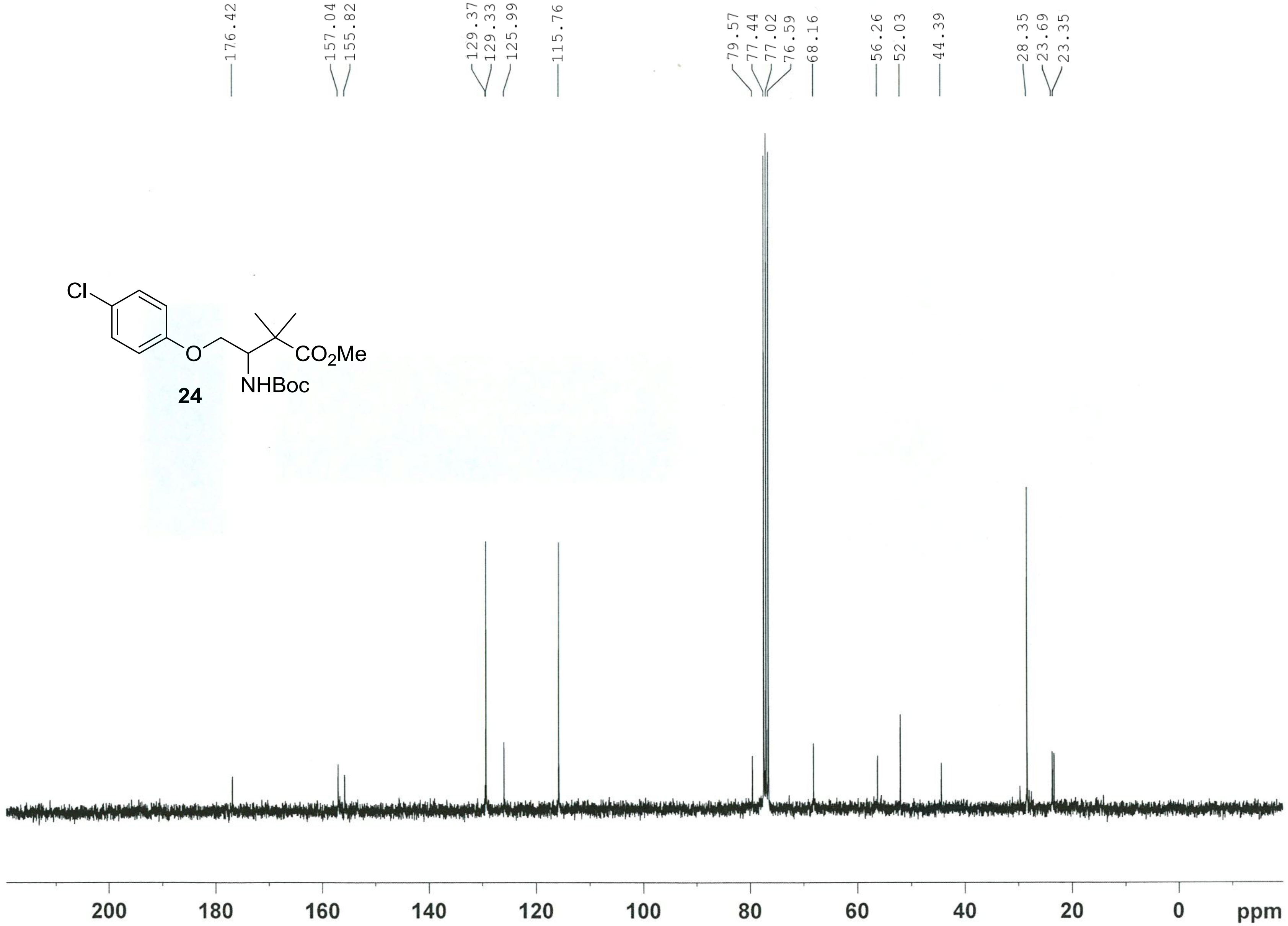

mdm-II-046 301b 1H NMR 12/26/08

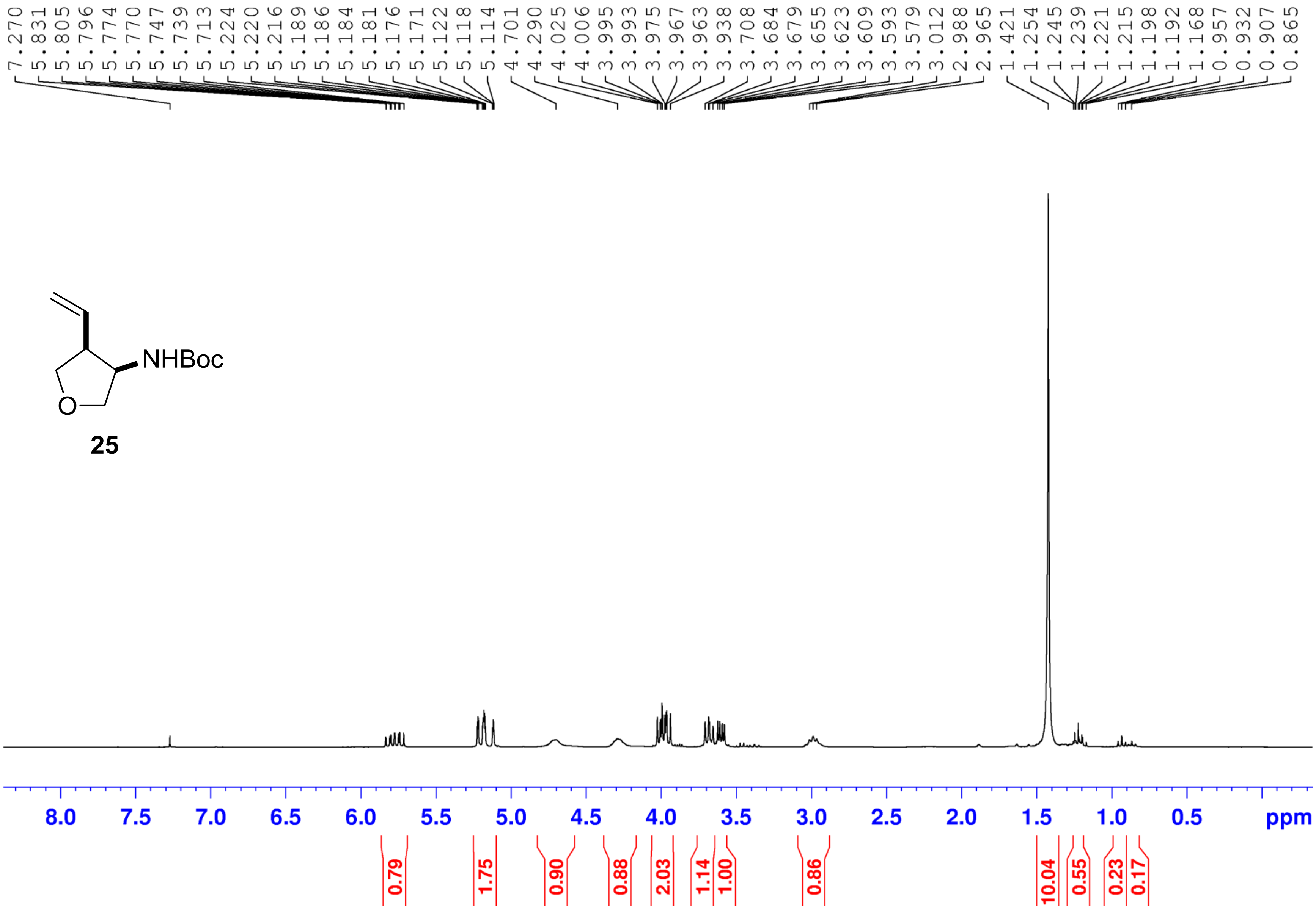

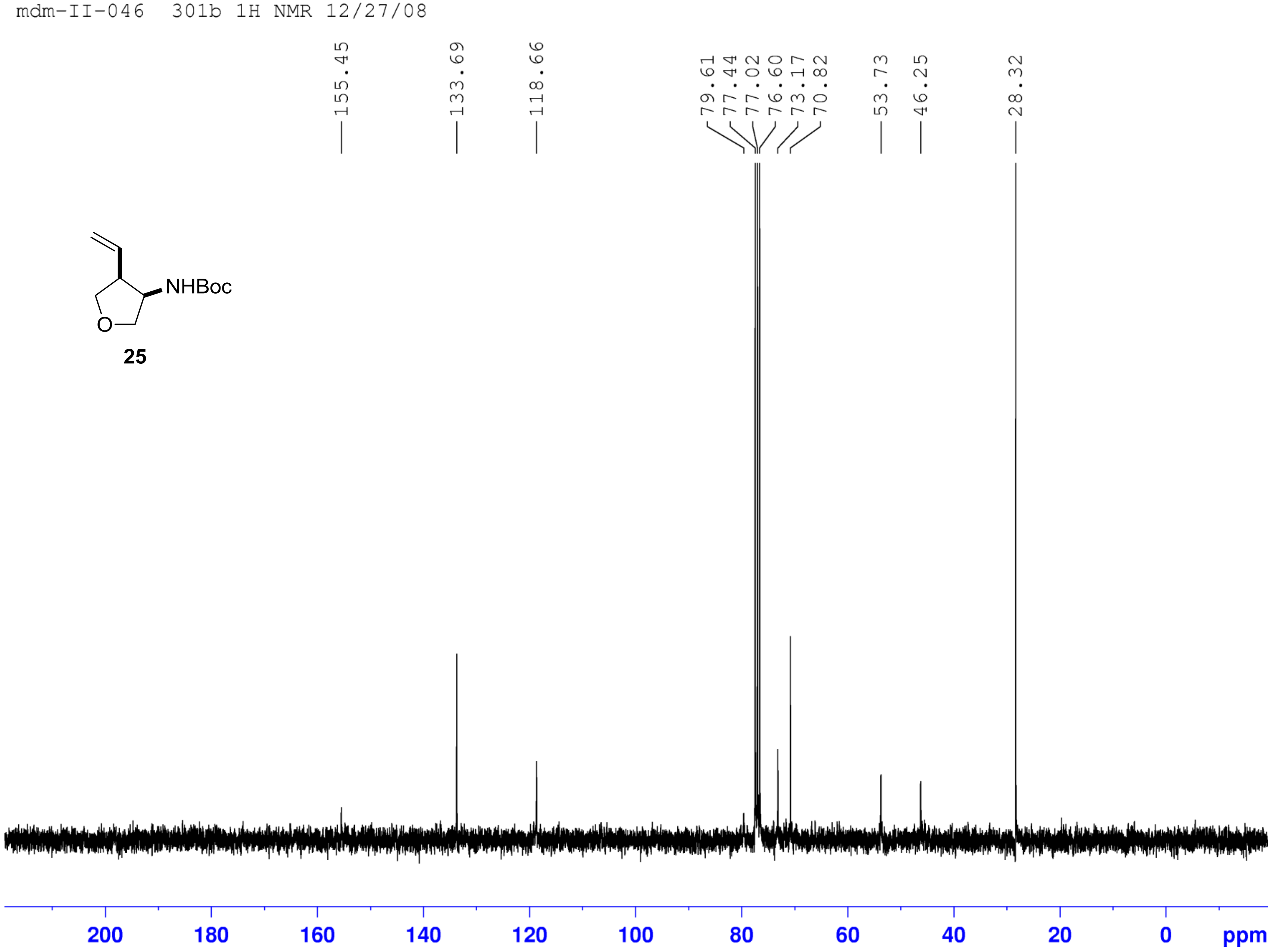
mdm-II-046 301b 1H NMR 12/27/08
155.45
133.69
118.66
79.61
77.44
77.02
76.60
73.17
70.82
53.73
46.25
28.32
NHBoc
O
25
200
180
160
140
120
100
80
60
40
20
0
ppm

mdm-II-074 301b 1H NMR 12/27/08

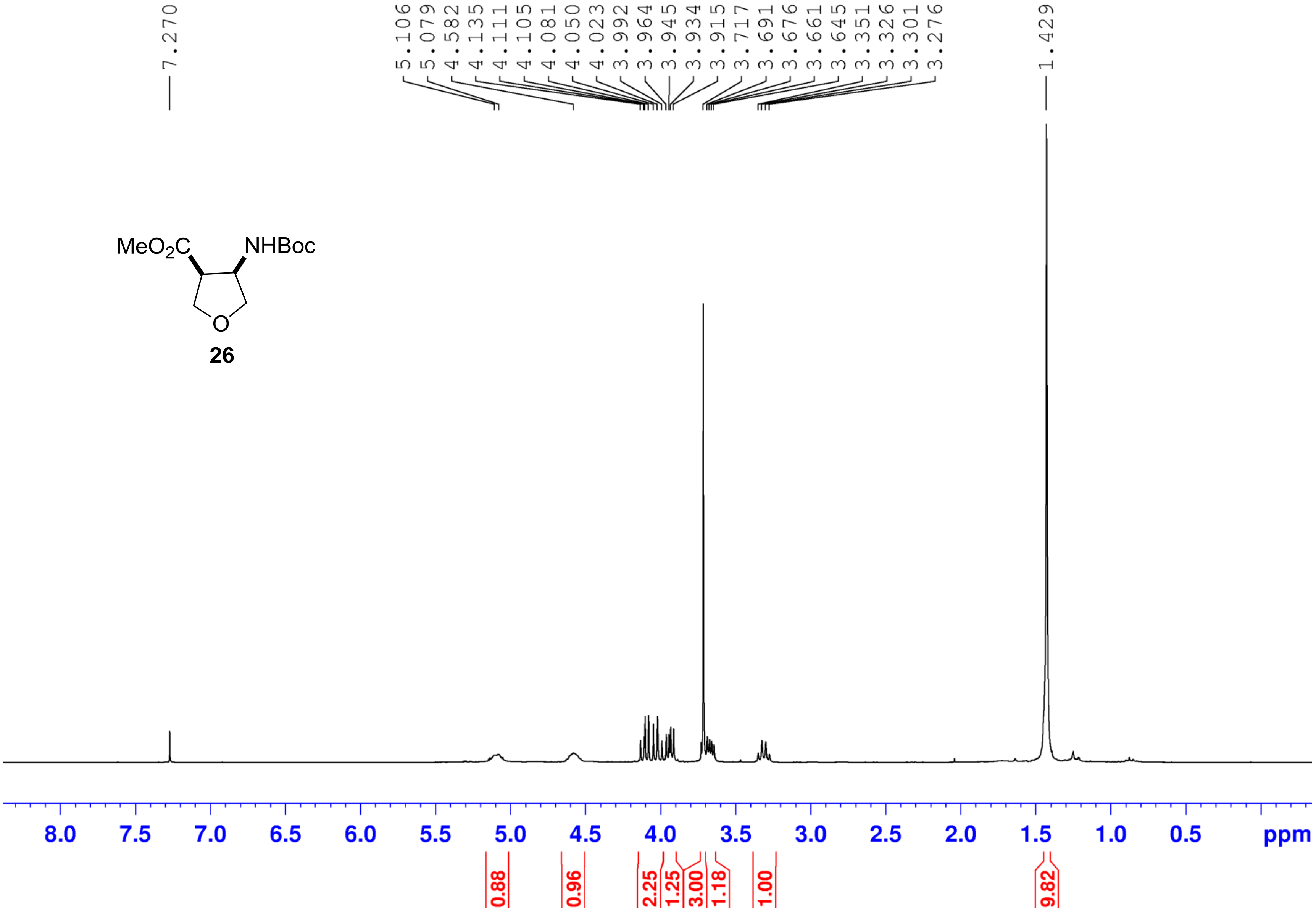


**26**

mdm-II-074 301b 13C NMR 12/27/08

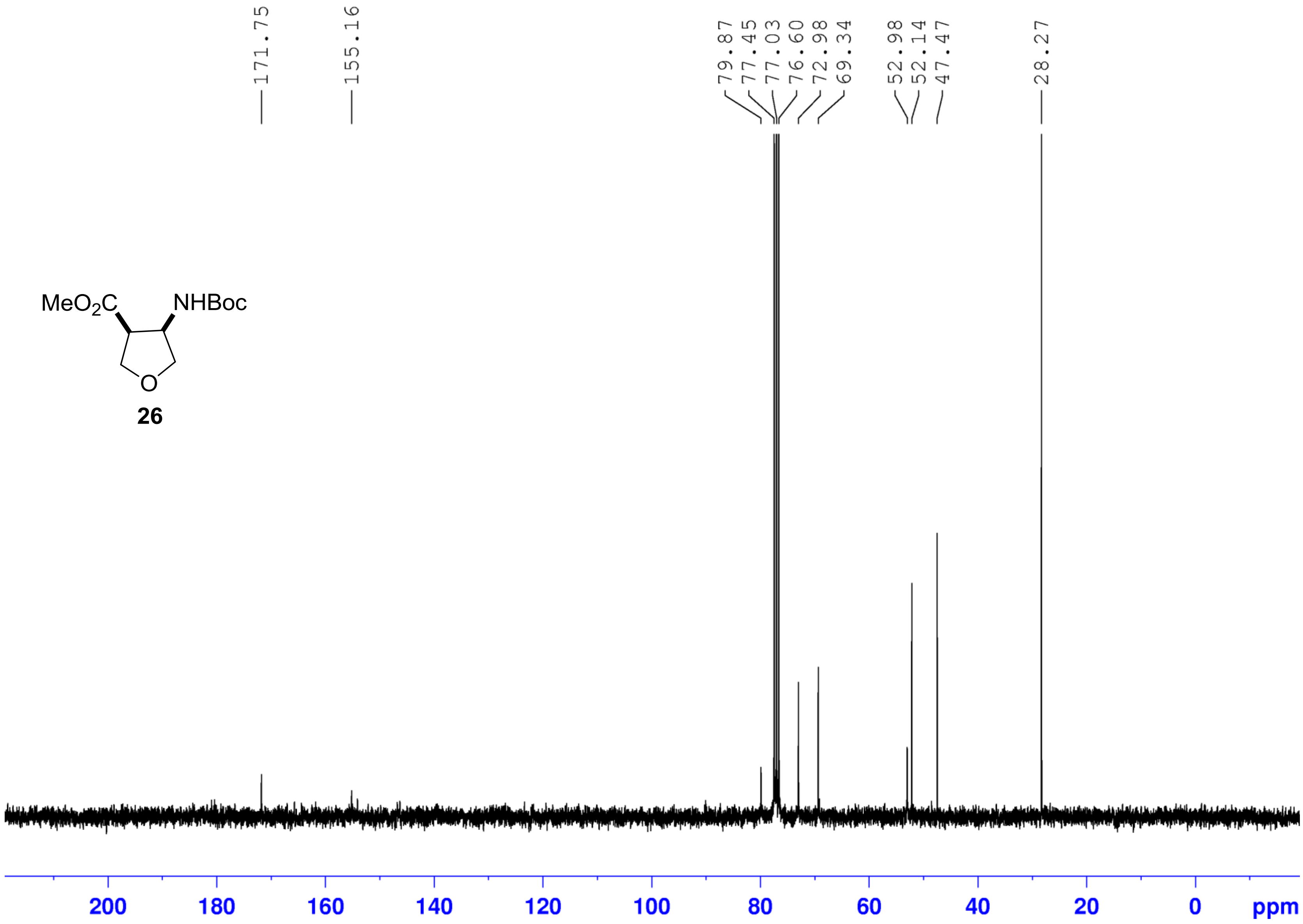

Supplement: File 1 — Experimental procedures and characterization details of synthesized compounds. [file Beilstein_J_Org_Chem-07-824-s001.pdf]
